# Supplementary material for: Interpretable systems biomarkers predict response to immune-checkpoint inhibitors
Source: Patterns (N Y). 2021 Jun 30;2(8):100293. doi: 10.1016/j.patter.2021.100293 (PMC8369166; doi:10.1016/j.patter.2021.100293)
Supplement: Document S1. Figures S1–S9 and Tables S5 and S6 [file mmc1.pdf]

Patterns, Volume *2*

Supplemental information

Interpretable systems biomarkers predict response to immune-checkpoint inhibitors

Óscar Lapuente-Santana, Maisa van Genderen, Peter A.J. Hilbers, Francesca Finotello, and Federica Eduati

SUPPLEMENTAL FIGURES

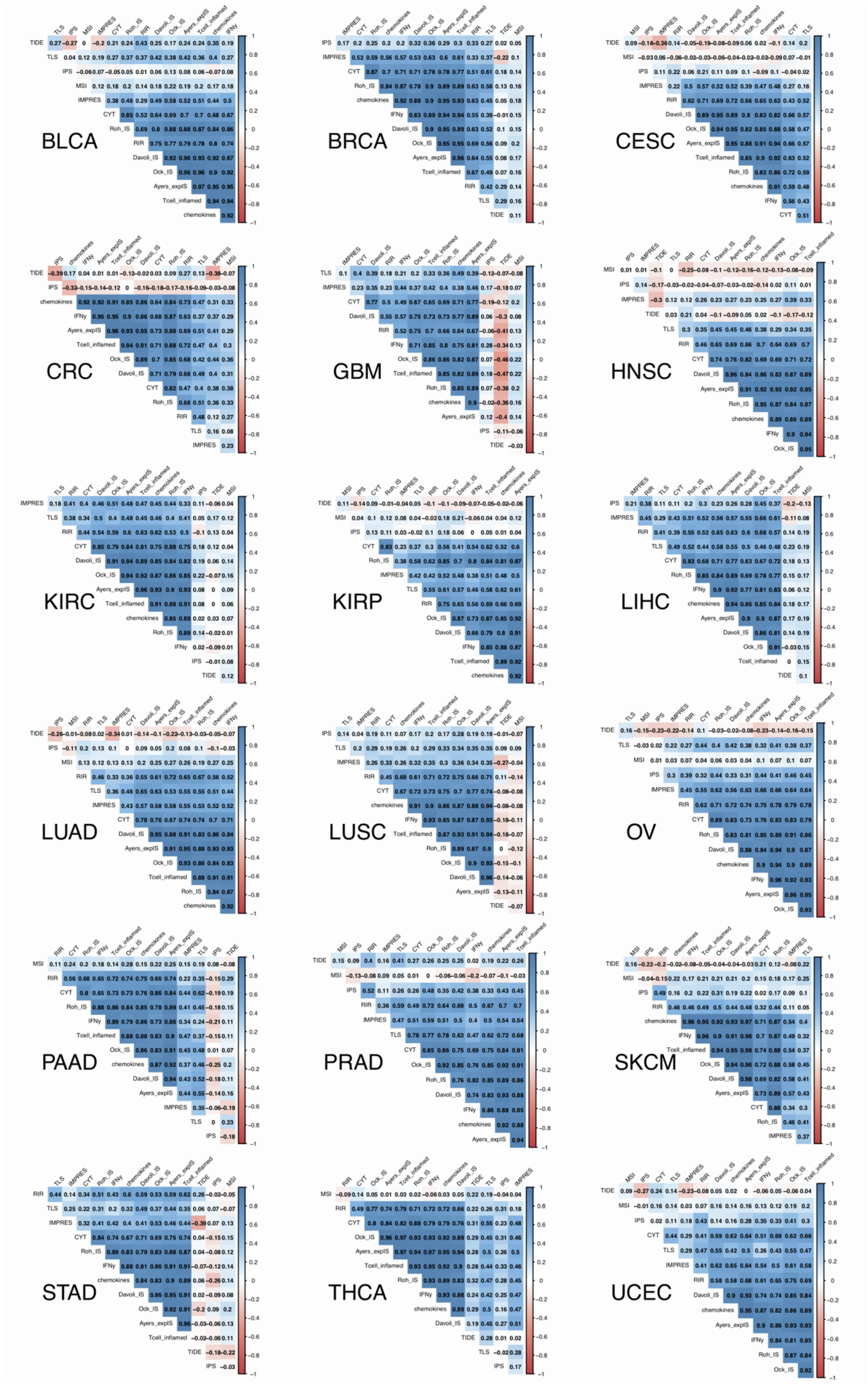

Figure S1. Cancer-specific Pearson correlations between the 14 proxies of immune response.

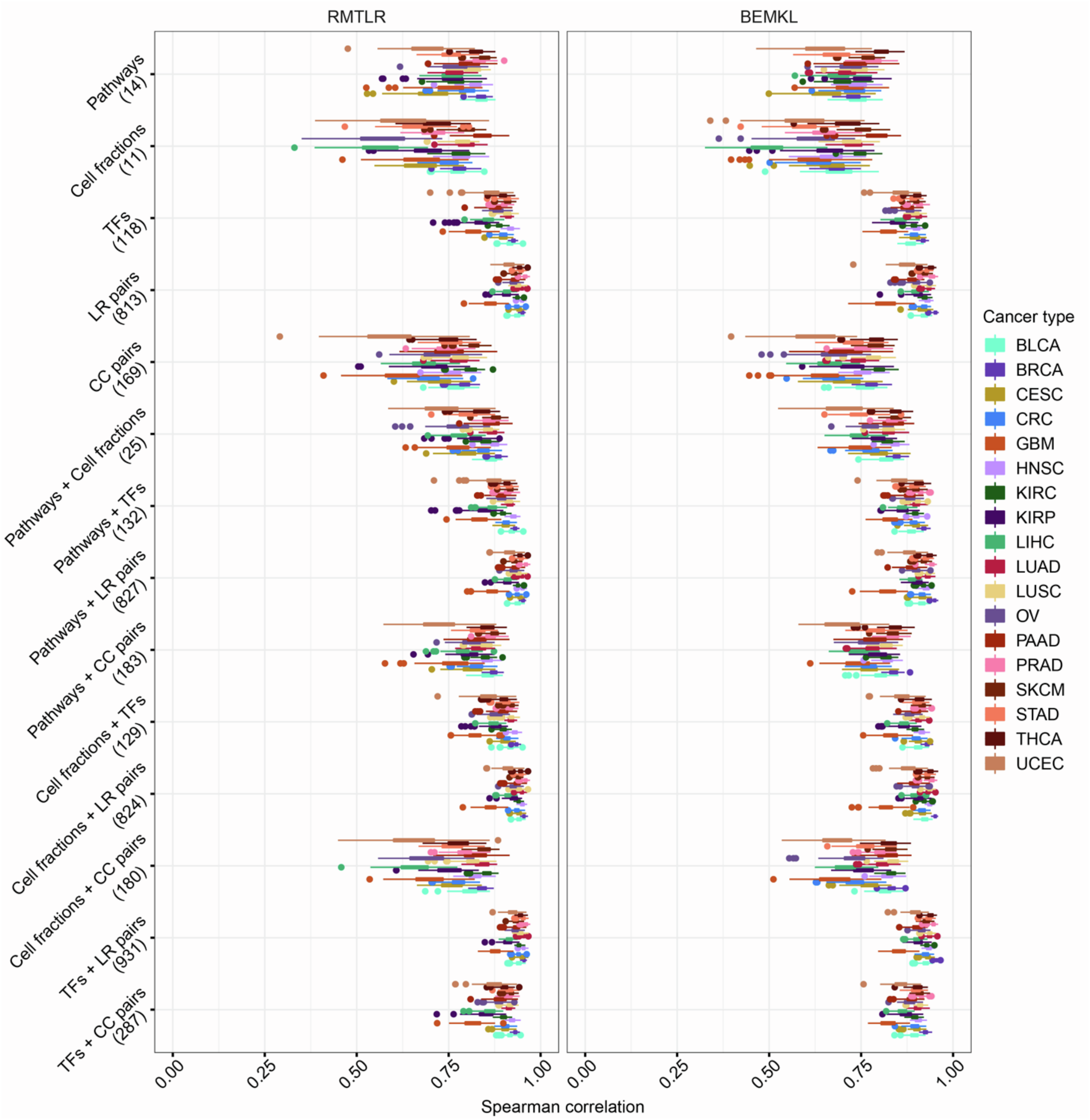

Figure S2. Randomized cross-validation performance of cancer-specific models trained on the TCGA patients. Models performances are shown for models trained separately for each system-based signature (single view) and for pairwise combinations of views. For each input data, the number of features is given in brackets.

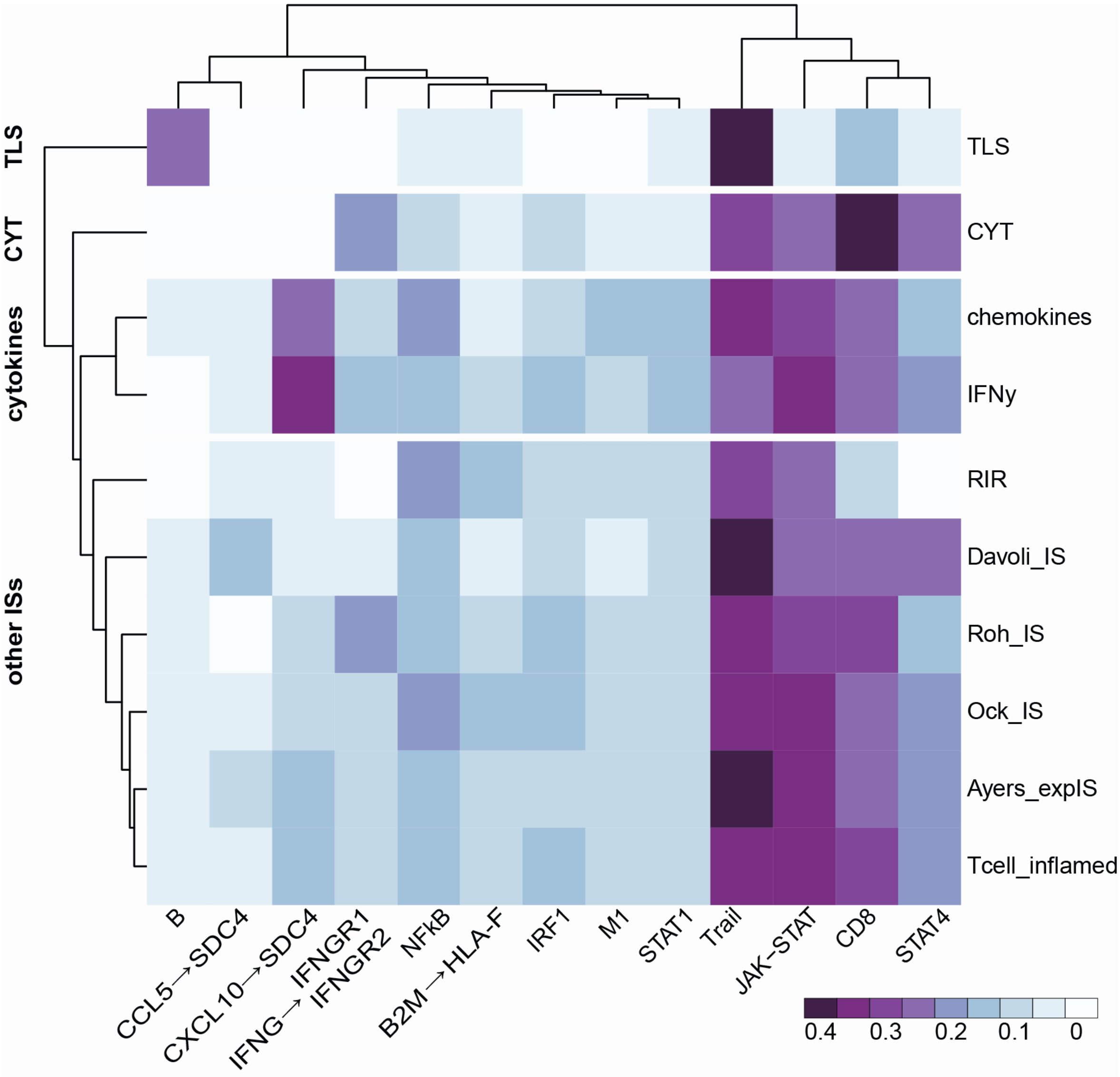

Figure S3. Heatmap showing the median across cancer types of the estimated weight of the features for each task. Only features with variance across tasks ≥ 0.0015 (top 1%) are shown in the heatmap. Tasks cluster in four main groups: 1. Tertiary lymphoid structures (TLS), 2. cytolytic activity (CYT), 3. cytokines related proxies (chemokines and IFNγ) , 4. all other immune signatures (ISs).

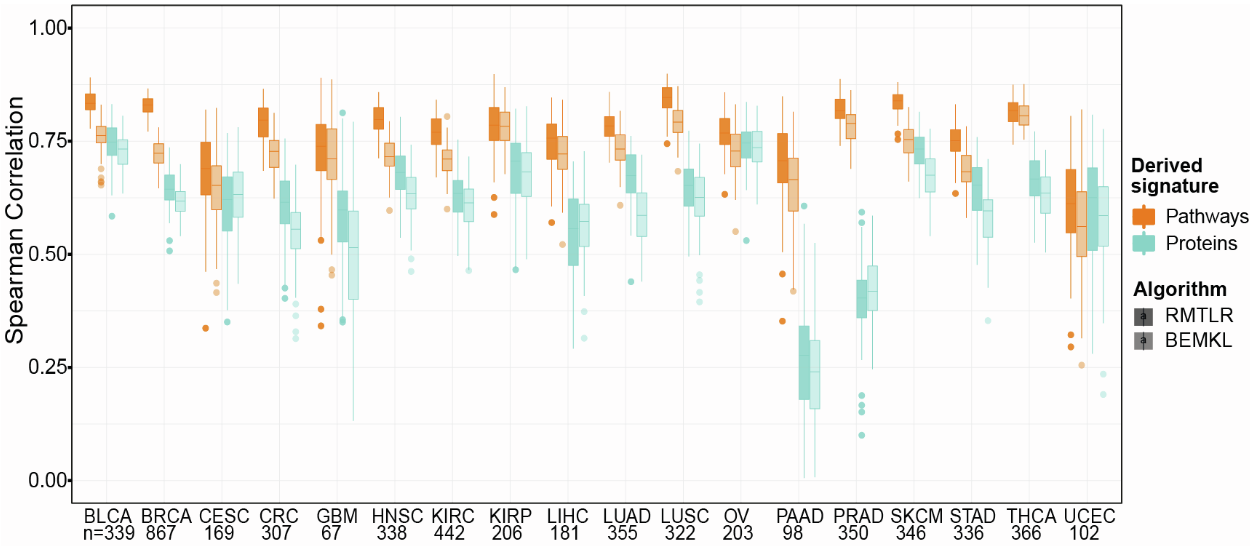

Figure S4. RMTLR and BEMKL cross-validation prediction performances for protein expression and pathway activity. Boxplots represent the distribution of prediction performances across the 100 randomized cross-validations on the TCGA data. The prediction performance is evaluated using Spearman correlation.

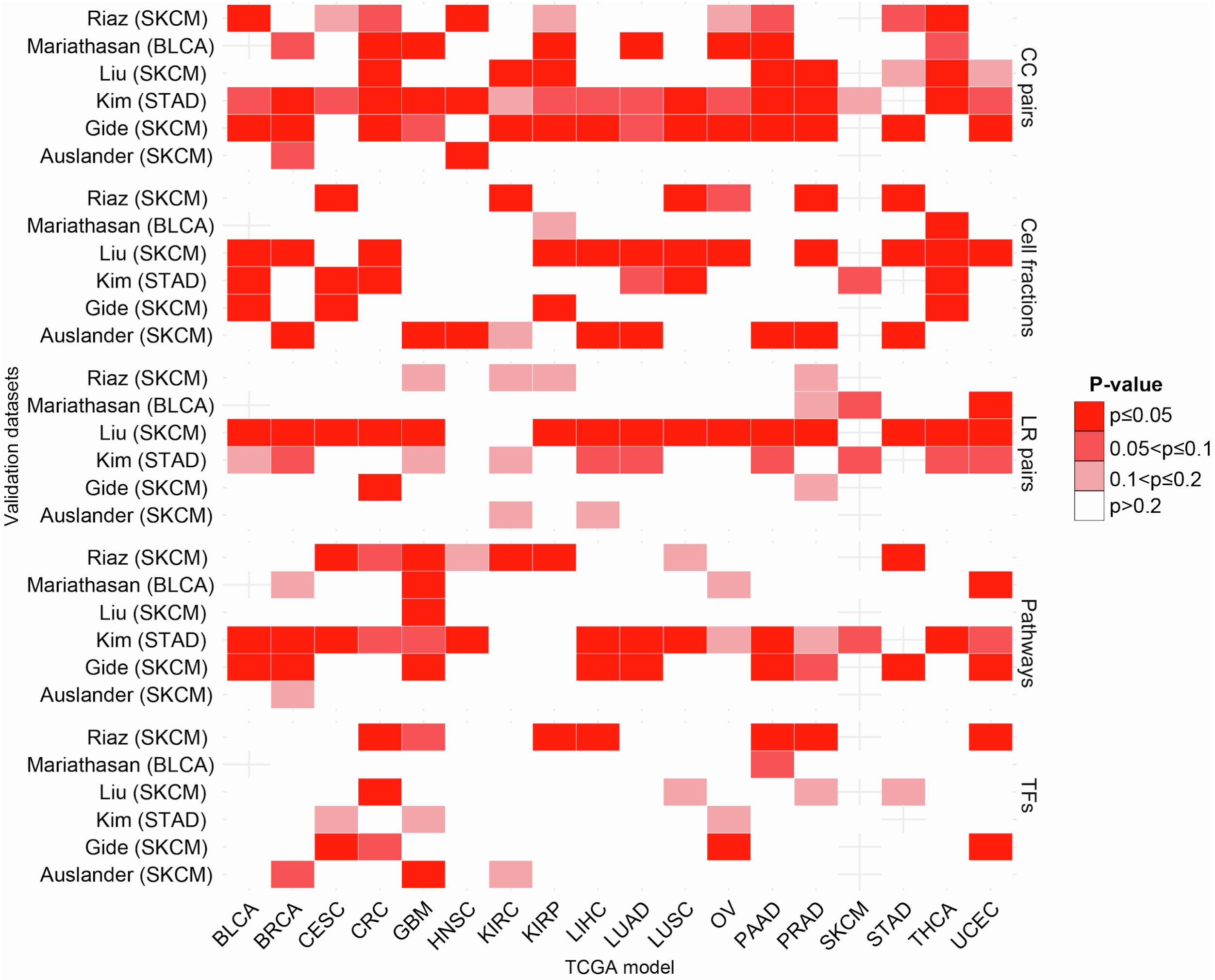

Figure S5. Comparison of prediction performance of cancer-type-specific models. The heatmap shows the p-value of the one-sided Wilcoxon-rank sum test used to assess if performances (AUC across tasks) obtained using the cancer-specific model for each dataset are better than performances obtained using the remaining 17 cancer-specific models (columns). This comparison was done for each view (different panels) and for each of the six datasets with known response to ICB (rows).

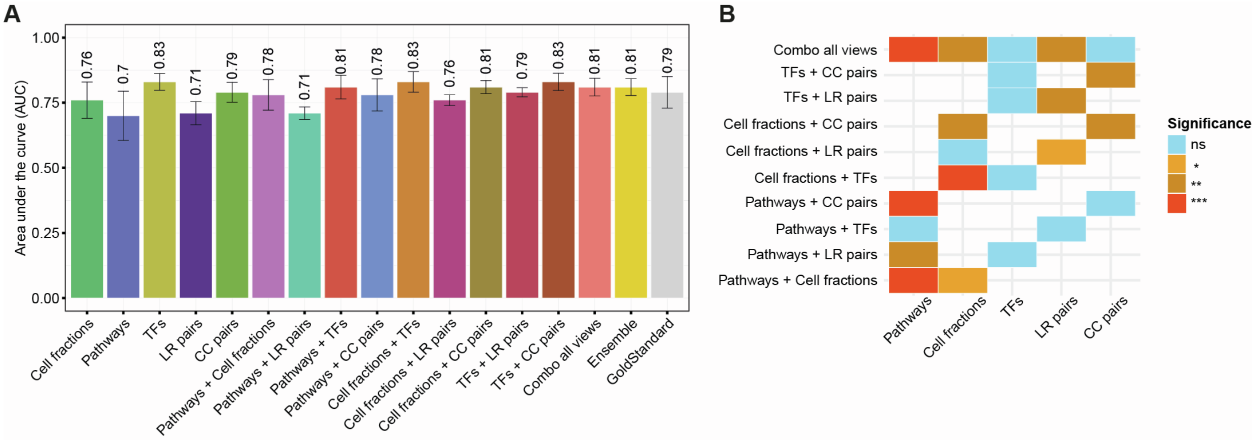

Figure S6. Prediction performances using BEMKL algorithm on the Auslander and Gide cohorts. (A) Area Under the Curve (AUC) values. Barplots represent the average AUC across tasks and error bars describe the corresponding standard deviation. (B) Performance comparison between single (x axis) and combined (y axis) views (one-side Wilcoxon signed-rank test). Statistical significance is indicated by colors according to the legend. The significant level (*p-value < 0.05, **p-value < 0.01, ***p-value < 0.001) indicates whether combining views improves the performance.

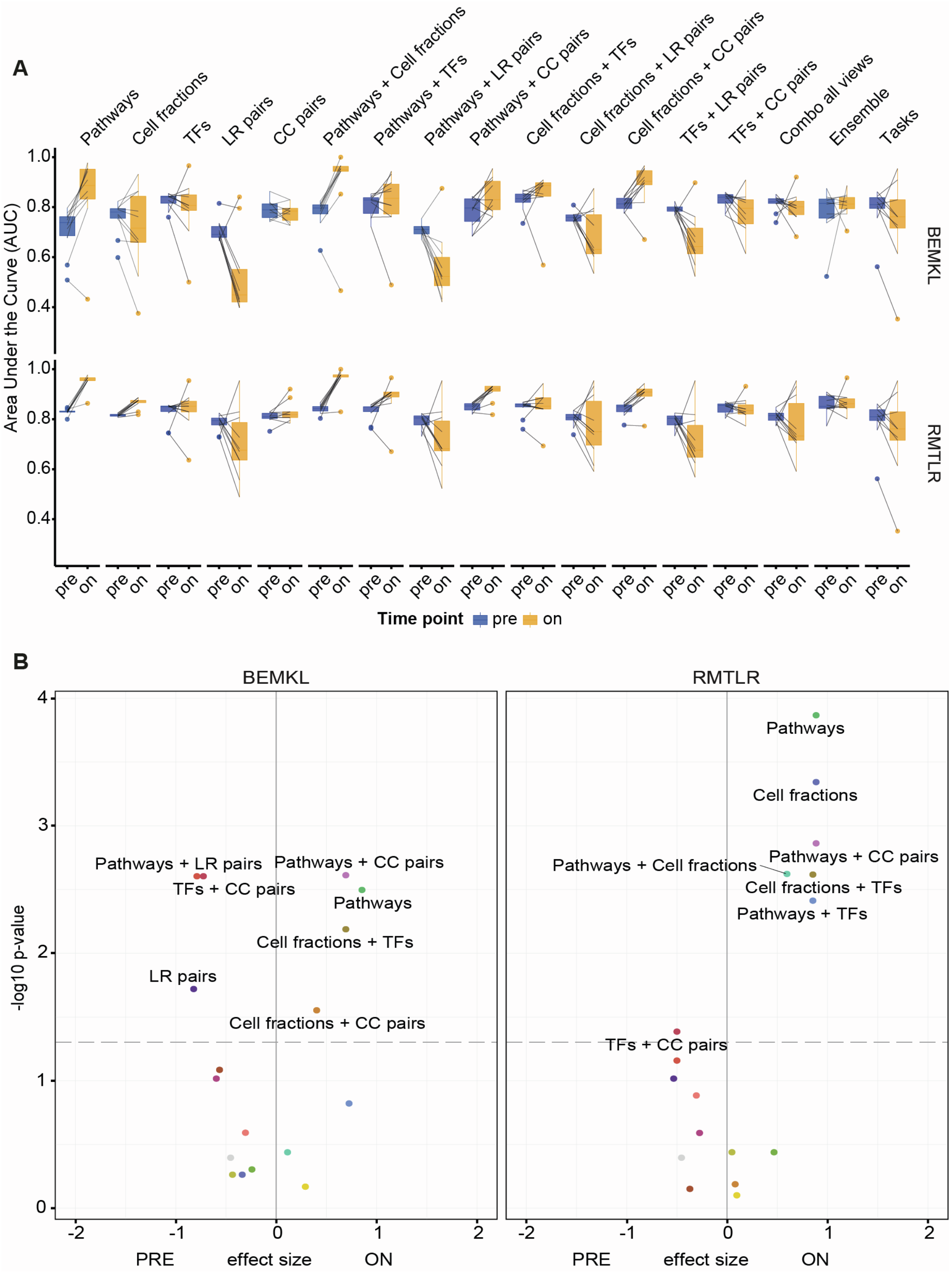

Figure S7. Comparison pre- vs on- treatment. (A) Comparison of Area Under the Curve (AUC) values for the different tasks computed on the pre-treatment (PRE) and on-treatment (ON) samples for the melanoma dataset. [1,2] Performances are shown for single views, pairwise combinations of views, combination of all views, average of single views predictions (ensemble), and all tasks (gold standard). (B) Volcano plots showing the statistical comparison of pre- vs on-treatment samples (two-sided Wilcoxon-rank sum test). Results are shown for both BEMKL and RMTLR algorithms.

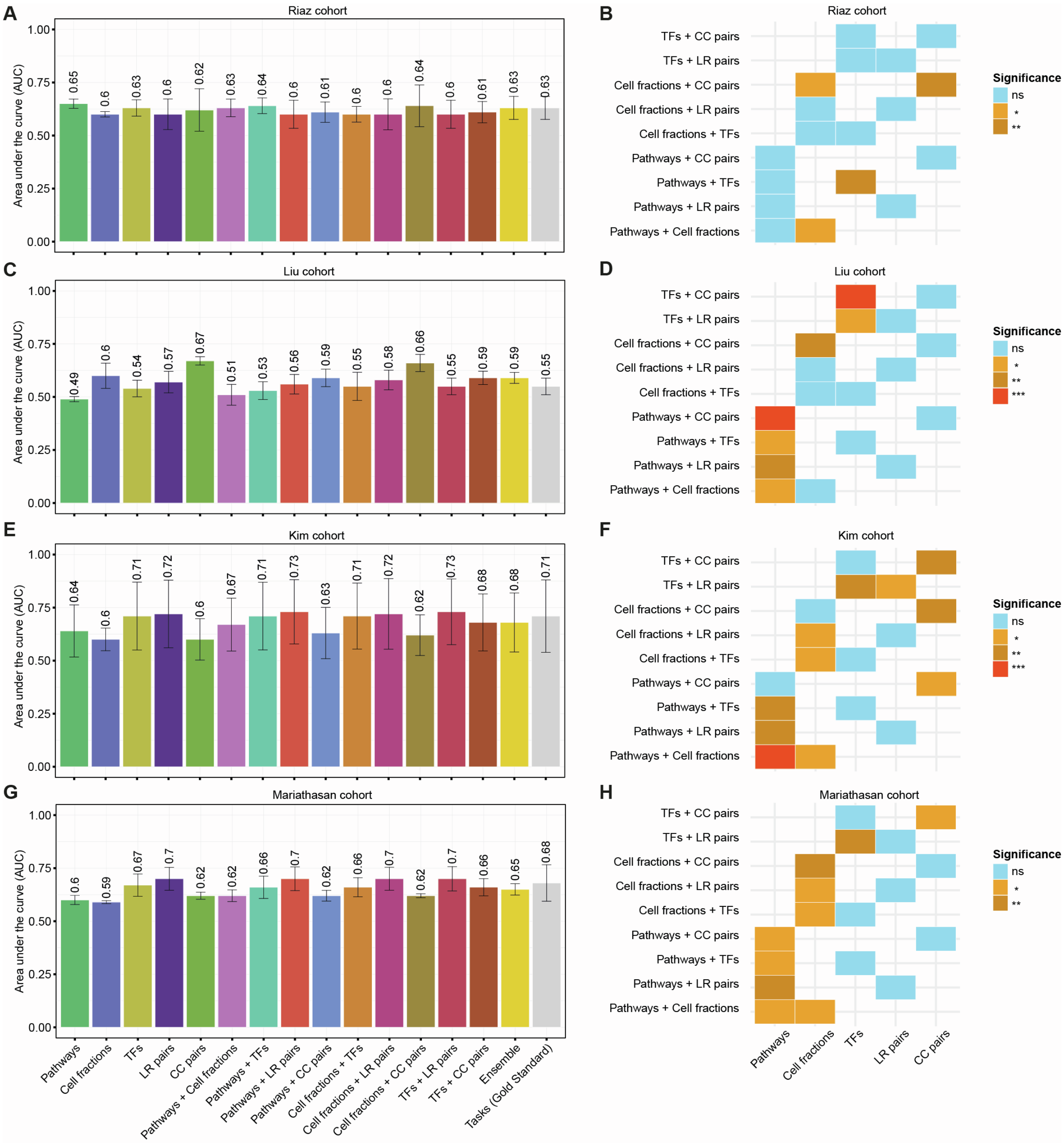

Figure S8. Overview of performances on the Riaz, Liu, Kim, and Mariathasan validation cohorts. Area Under the Curve (AUC) values for the (A) Riaz, (C) Liu, (E) Kim and (G) Mariathasan cohorts using the RMTLR algorithm. Performance comparison between single (x axis) and combined (y axis) views for (B) Riaz, (D) Liu, (F) Kim and (H) Mariathasan cohorts (one-side Wilcoxon signed-rank test). Statistical significance is indicated by colors according to the legend. The significant level (*p-value < 0.05, **p-value < 0.01, ***p-value < 0.001) indicates whether combining views improves the performance.

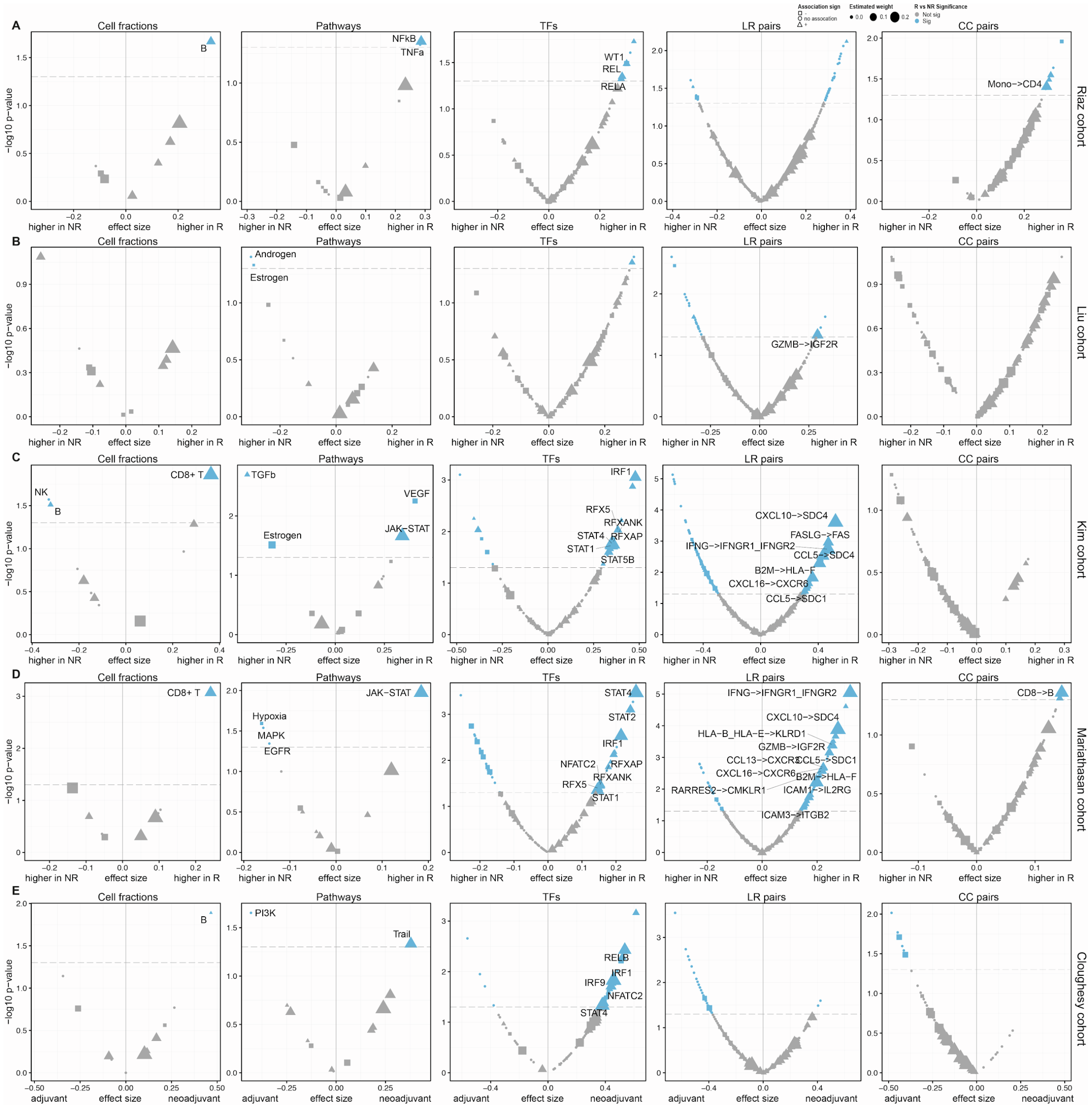

Figure S9. Comparison of biomarkers in responders vs non-responders. Volcano plots for systems biomarkers of the immune response from the (A) Riaz, (B) Liu, (C) Kim, (D) Mariathsan and (E) Cloughesy cohorts comparing non-responder (NR) and responder (R) patients. In the Cloughesy cohort, volcano plots compare systems biomarkers in adjuvant vs neoadjuvant anti-PD-1 therapy patients (two-sided Wilcoxon-rank sum test). Significant biomarkers (p-value < 0.05) are shown in blue. Biomarkers are drawn according to their corresponding sign (shape) and weight (size) obtained during model training. Labels are reported for the top 15 cancer-specific biomarkers *(based on the association with the tasks)* that are significantly different between R and NR.

SUPPLEMENTAL TABLES

Table S5. Overview of validation datasets.

Original study	Cancer type	TMB available	Prior therapies	Biopsy	Samples used	R and NR	RNA-seq fastq files
Auslander [1]	Melanoma (Metastasis)	NO	Therapy naive	Fresh-frozen	PD-1:<br>- Pre: n=9 (R=1, NR=8)<br>- On: n=17 (R=0, NR=17)	As reported in publication.	BioProject ID: PRJNA476140
Gide [2]	Melanoma (Metastasis)	NO	BRAFi	FFPE	PD-1:<br>- Pre: n=41 (CR=4, PR=15, PD=16, SD=6)<br>- On: n=9 (CR=0, PR=4, PD=4, SD=1)	R= CR, PR<br>NR= SD, PD	BioProject ID: PRJEB23709
Kim [3]	Gastric cancer (Metastasis)	YES	Prior failure of at least 1 line of chemotherapy (platinum)	Fresh-frozen	Pre: n=45 (CR=3, PR=9, PD=18, SD=15)	R= CR, PR<br>NR= SD, PD	BioProject ID: PRJEB25780
Riaz [4]	Advanced melanoma	YES	Nivo naive<br>Nivo prog	FFPE	Pre: n=45 (CR=3, PR=7, PD=23, SD=16)	R= CR, PR<br>NR= SD, PD	BioProject ID: PRJNA356761
Liu [5]	Melanoma (Metastasis)	YES	Prior MAPKTx<br>Therapy naive	FFPE	Pre: n=46 (CR=9, MR=1, PR=10, PD=19, SD=7)	R= CR, PR<br>NR= SD, PD	Supplementary files of the study
Mariathasan [6]	Bladder cancer	YES	Platinum based chemotherapy<br>Therapy naive	FFPE	Pre: n=192 (CR=25, PD=167)	R= CR<br>NR= PD	IMVigor210Biologies R package
Cloughesy [7]	Recurrent glioblastoma	NO	Previous first-line therapy with at least radiotherapy	Fresh-frozen	Pre: n=28<br>adjuvant anti-PD-1 = 15<br>neoadjuvant anti-PD-1 = 13	NA	BioProject ID: PRJNA498500

FFPE: Formalin-fixed paraffin-embedded
CR: Complete Responder; PR: Partial Responder; PD: Progressive Disease; SD: Stable Disease
R: Responder; NR: Non-responder

Table S6. List of cells included in the TME network.

Aggregate cell type	Cells in the literature	Cell types in network	Derivation
**Adipocytes**	Adipocytes [8,9]	Mature Adipocyte<br>Adipocyte Omental	
**B-cells**	B-cell [10]<br>CD20 B-cell [9,11]<br>CD19 B-cell [9]	CD19+ B cells	
**CD4+ T cells**	CD4+ T helper cells [8–10]<br>CD4+ T regulatory cells [8,10]	CD4+ T cells<br>CD4+CD25+CD45RA+ naive regulatory T-cells<br>CD4+CD25+CD45RA- memory regulatory T-cells<br>CD4+CD25-CD45RA+ naive conventional T-cells<br>CD4+CD25-CD45RA+ memory conventional T-cells	CD4+ NR T cells<br>CD4+ MR T cells<br>CD4+ NC T cells<br>CD4+ MC T cells
**CD8+ T cells**	CD8+ cytotoxic T cells [8–11]	CD8+ T cells	
**Dendritic cells**	Dendritic cell [8–11]	Dendritic Plasmacytoid<br>Dendritic Monocyte Immature derived	Dendritic Monocyte Id
**Endothelial cells**	Endothelial cells [10,11]<br>Vascular endothelial cells [8]<br>Lymphatic endothelial cells [8]	Endothelial Microvascular<br>Endothelial Lymphatic	
**Fibroblasts**	Fibroblasts [8–11]	Fibroblast Lymphatic<br>Fibroblast Skin Normal	
**Macrophages**	M1 macrophage [8–12]<br>M2 macrophage [8–10,12]	Macrophage Monocyte derived	Macrophage Monocyte
**Mast Cells**	Mast cells [9–11]	Mast cells<br>Mast cells stimulated	
**Monocytes Myeloid**	Myeloid derived suppressor cells [10,11]<br>Monocytes [9,11,12]	CD14+ Monocytes<br>CD14+CD16+ Monocytes<br>CD14+CD16- Monocytes<br>CD14-CD16+ Monocytes	
**NK Cells**	Natural killer cells [8–11]<br>Natural killer T-cells [8]	NK cells	
**Neutrophils**	Neutrophils [8,11]	Neutrophils	

SUPPLEMENTAL REFERENCES

1. Auslander, N., Zhang, G., Lee, J.S., Frederick, D.T., Miao, B., Moll, T., Tian, T., Wei, Z., Madan, S., Sullivan, R.J., et al. (2018). Robust prediction of response to immune checkpoint blockade therapy in metastatic melanoma. Nat. Med. *24*, 1545–1549.

2. Gide, T.N., Quek, C., Menzies, A.M., Tasker, A.T., Shang, P., Holst, J., Madore, J., Lim, S.Y., Velickovic, R., Wongchenko, M., et al. (2019). Distinct Immune Cell Populations Define Response to Anti-PD-1 Monotherapy and Anti-PD-1/Anti-CTLA-4 Combined Therapy. Cancer Cell *35*, 238–255.e6.

3. Kim, S.T., Cristescu, R., Bass, A.J., Kim, K.-M., Odegaard, J.I., Kim, K., Liu, X.Q., Sher, X., Jung, H., Lee, M., et al. (2018). Comprehensive molecular characterization of clinical responses to PD-1 inhibition in metastatic gastric cancer. Nat. Med. *24*, 1449–1458.

4. Riaz, N., Havel, J.J., Makarov, V., Desrichard, A., Urba, W.J., Sims, J.S., Hodi, F.S., Martín-Algarra, S., Mandal, R., Sharfman, W.H., et al. (2017). Tumor and Microenvironment Evolution during Immunotherapy with Nivolumab. Cell *171*, 934–949.e16.

5. Liu, D., Schilling, B., Liu, D., Sucker, A., Livingstone, E., Jerby-Arnon, L., Zimmer, L., Gutzmer, R., Satzger, I., Loquai, C., et al. (2019). Integrative molecular and clinical modeling of clinical outcomes to PD1 blockade in patients with metastatic melanoma. Nat. Med. *25*, 1916–1927.

6. Mariathasan, S., Turley, S.J., Nickles, D., Castiglioni, A., Yuen, K., Wang, Y., Kadel, E.E., III, Koeppen, H., Astarita, J.L., Cubas, R., et al. (2018). TGFβ attenuates tumour response to PD-L1 blockade by contributing to exclusion of T cells. Nature *554*, 544–548.

7. Cloughesy, T.F., Mochizuki, A.Y., Orpilla, J.R., Hugo, W., Lee, A.H., Davidson, T.B., Wang, A.C., Ellingson, B.M., Rytlewski, J.A., Sanders, C.M., et al. (2019). Neoadjuvant anti-PD-1 immunotherapy promotes a survival benefit with intratumoral and systemic immune responses in recurrent glioblastoma. Nat. Med. *25*, 477–486.

8. Balkwill, F.R., Capasso, M., and Hagemann, T. (2012). The tumor microenvironment at a glance. J. Cell Sci. *125*, 5591–5596.

9. Wang, M., Zhao, J., Zhang, L., Wei, F., Lian, Y., Wu, Y., Gong, Z., Zhang, S., Zhou, J., Cao, K., et al. (2017). Role of tumor microenvironment in tumorigenesis. J. Cancer *8*, 761–773.

10. Fridman, W.H., Pagès, F., Sautès-Fridman, C., and Galon, J. (2012). The immune contexture in human tumours: impact on clinical outcome. Nat. Rev. Cancer *12*, 298–306.

11. Fridman, W.H., Zitvogel, L., Sautès-Fridman, C., and Kroemer, G. (2017). The immune contexture in cancer prognosis and treatment. Nat. Rev. Clin. Oncol. *14*, 717–734.

12. Richards, D.M., Hettinger, J., and Feuerer, M. (2013). Monocytes and Macrophages in Cancer: Development and Functions. Cancer Microenvironment *6*, 179–191.
