# Supplementary material for: Interpretable systems biomarkers predict response to immune-checkpoint inhibitors
Source: Patterns (N Y). 2021 Jun 30;2(8):100293. doi: 10.1016/j.patter.2021.100293 (PMC8369166; doi:10.1016/j.patter.2021.100293)
Supplement: Document S2. Article plus supplemental information [file mmc6.pdf]

Patterns

Article

Interpretable systems biomarkers predict response to immune-checkpoint inhibitors

Graphical abstract

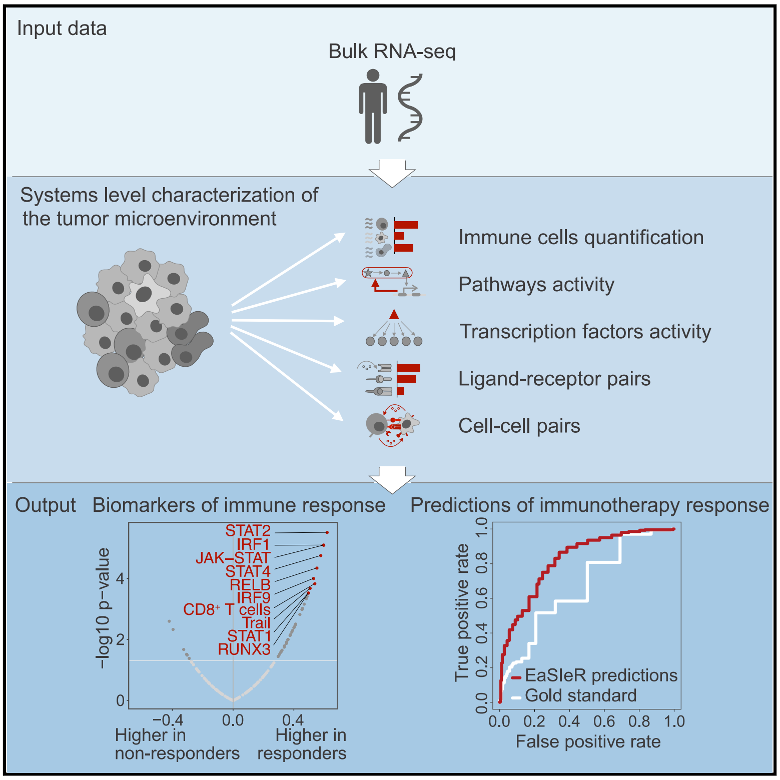

Highlights

- Characterization of the tumor microenvironment from RNA-seq and prior knowledge
- Multi-task machine-learning models for predicting antitumor immune responses
- Identification of cancer-type-specific, interpretable biomarkers of immune responses
- EaSIeR is a tool to predict biomarker-based immunotherapy response from RNA-seq

Authors

Óscar Lapuente-Santana, Maisa van Genderen, Peter A.J. Hilbers, Francesca Finotello, Federica Eduati

Correspondence

f.eduati@tue.nl

In brief

Immunotherapy with immune-checkpoint blockers (ICBs) is a promising treatment for different cancer types providing a long-lasting clinical benefit. However, only a fraction of cancer patients respond to this treatment. Biomarkers to effectively predict patients' responses are needed. This article addresses this problem using a systems approach to characterize antitumor immune response taking into account the tumor microenvironment as a whole. Using interpretable machine learning, we derive mechanistic biomarkers and cancer-specific models of response predicting patients' response to ICB.

Lapuente-Santana et al., 2021, Patterns *2*, 100293
August 13, 2021 © 2021 The Author(s).
https://doi.org/10.1016/j.patter.2021.100293

Patterns

CellPress
OPEN ACCESS

Article

Interpretable systems biomarkers predict response to immune-checkpoint inhibitors

Óscar Lapuente-Santana,[1] Maisa van Genderen,[1] Peter A.J. Hilbers,[1] Francesca Finotello,[2] and Federica Eduati[1,3,4,*]
[1]Department of Biomedical Engineering, Eindhoven University of Technology, 5612 AZ Eindhoven, the Netherlands
[2]Biocenter, Institute of Bioinformatics, Medical University of Innsbruck, 6020 Innsbruck, Austria
[3]Institute for Complex Molecular Systems, Eindhoven University of Technology, 5612 AZ Eindhoven, the Netherlands
[4]Lead contact
*Correspondence: f.eduati@tue.nl
https://doi.org/10.1016/j.patter.2021.100293

THE BIGGER PICTURE Identification of biomarkers of response to immune-checkpoint blockers (ICBs) is a major challenge in immuno-oncology. Several studies have focused on specific mechanisms orchestrating antitumor immune responses, significantly improving our understanding of the functioning of constitutive parts of the tumor microenvironment (TME) and their relevance for the success of ICBs. However, tumors are complex systems, and understanding the immune response in the TME requires holistic strategies. To overcome this challenge, we derived higher-level representation of the TME by integrating RNA-sequencing (RNA-seq) data with different types of prior knowledge. We used interpretable machine learning to extract from these structured data mechanistic biomarkers of antitumor immune responses that effectively predicted patients' response to ICBs. Our approach has potential for clinical application as it requires only patients' tumor RNA-seq data to quantify biomarkers and predict patients' likelihood of response to ICB.

1 2 **3** 4 5 **Development/Pre-production:** Data science output has been rolled out/validated across multiple domains/problems

SUMMARY

Cancer cells can leverage several cell-intrinsic and -extrinsic mechanisms to escape immune system recognition. The inherent complexity of the tumor microenvironment, with its multicellular and dynamic nature, poses great challenges for the extraction of biomarkers of immune response and immunotherapy efficacy. Here, we use RNA-sequencing (RNA-seq) data combined with different sources of prior knowledge to derive system-based signatures of the tumor microenvironment, quantifying immune-cell composition and intra- and intercellular communications. We applied multi-task learning to these signatures to predict different hallmarks of immune responses and derive cancer-type-specific models based on interpretable systems biomarkers. By applying our models to independent RNA-seq data from cancer patients treated with PD-1/PD-L1 inhibitors, we demonstrated that our method to Estimate Systems Immune Response (EaSIeR) accurately predicts therapeutic outcome. We anticipate that EaSIeR will be a valuable tool to provide a holistic description of immune responses in complex and dynamic systems such as tumors using available RNA-seq data.

INTRODUCTION

In the past few years, immunotherapy has revolutionized cancer treatment, especially based on antibodies targeting immune checkpoints, such as the cytotoxic T-lymphocyte-associated protein 4 (CTLA-4), programmed cell death protein (PD-1), or its ligand (PD-L1).[1] Immune-checkpoint blockers (ICBs) boost the patient's immune system to effectively recognize and attack cancer cells. In different cancer types, patients treated with these immune-based therapies have shown promising results, especially in terms of long-term patient survival and even curative potential. Despite this fact, just a minority of the patients achieve complete response. In addition, high immunological toxicity[2,3] and considerable costs (>US$100,000 per patient per year)[4] are other challenges for ICB therapy. That is why biomarkers are indispensable for selecting potential responders and sparing unnecessary and potentially harmful treatments to patients who are unlikely to respond to ICBs.[5]

Different mechanisms in the tumor microenvironment (TME) are involved in mediating the immune response and affect the

This is an open access article under the CC BY license (http://creativecommons.org/licenses/by/4.0/).

efficacy of ICB therapy.[6] A first important aspect is the cell-type composition of the TME. Different types of TME cells, and especially immune cells, can have a pro- or antitumor role in regulating cancer progression and response to treatment.[7] A key role in antitumor response is played by effector T cells: their phenotype, abundance, and localization within the TME are major determinants of immunotherapy success.[8] Another important aspect is the inter- and intracellular regulation of cellular functions that are responsible for shaping the anticancer immune response. Signals from outside the cells are processed by intracellular signaling pathways leading to changes in transcription factor (TF) activity and gene expression. Intracellular regulatory networks of tumor cells are involved in innate (endogenous, due to mutations) and adaptive (due to exogenous stimulation) mechanisms that tumor cells exploit to resist immune attack.[9] This can be accomplished by different mechanisms, such as the upregulation of immune checkpoints,[10] reduced release of inflammatory cytokines,[11,12] or impaired antigen presentation by the major histocompatibility complex (MHC).[13] These are all important mechanisms that cancer cells use to communicate with surrounding cells. More in general, ligand-receptor (LR) interactions regulate cell-cell (CC) communication between all the cells in the microenvironment, including tumor cells, immune cells, and fibroblasts, and finely regulate tumor characteristics and antitumor immune responses.[12,14,15]

All these aspects should be taken into account to provide a comprehensive description of the TME. A holistic approach to derive biomarkers of immune response can inform clinicians on the efficacy of ICB therapy in individual patients.[6,16] Different emerging omics technologies allow one to take snapshots of the TME in bulk tumors, in single cells, or from images of tumor tissue slides. The combination of these cutting-edge technologies with new computational tools holds great potential to provide a complete picture of the TME, shedding light on how complex cellular and intercellular mechanisms orchestrate the immune response.[17,18] However, such technologies are not yet widely available, and computational tools to fully exploit their potential are still in their infancy. To improve precision medicine, we urgently need different approaches to derive a comprehensive description of the TME and how it regulates immune response in individual patients, using currently available patient data. Bulk RNA sequencing (RNA-seq) has become the *de facto* method to quantify transcriptome-wide gene expression[19] and is increasingly available, not only through public databases and collaborative efforts like The Cancer Genome Atlas (TCGA),[20] but also in small-to-midsize laboratories, as well as in the clinics.[21]

Here, we describe an approach based on RNA-seq data combined with different types of prior knowledge to derive a holistic description of the TME. In particular we use validated computational methods to quantify tumor-infiltrating immune cells,[22] activity of intracellular signaling and TFs,[23,24] and extent of intercellular communication[14] from bulk-tumor RNA-seq data. Using multi-task machine learning algorithms, we aim to assess how these system-based signatures of the TME are associated with 14 different transcriptome-based predictors of anticancer immune responses (Table S1), which model different hallmarks of response to ICB therapy.

By training machine learning models on RNA-seq data from 7,550 patients' data across 18 solid cancers generated by TCGA, we identified predictive, interpretable system-based biomarkers of immune response in a cancer-type-specific fashion. This integrative approach allowed us to identify several biomarkers that are known to be associated with immune response and response to ICBs, as well as new candidates for future follow-up studies. In addition, we show how the derived system-based biomarkers of immune response are able to predict response to ICB therapy in independent datasets of cancer patients treated with anti-PD-1/anti-PD-L1. This proposed computational framework is provided as a tool called Estimate Systems Immune Response (EaSIeR) that can be applied to bulk-tumor RNA-seq data to investigate mechanistic biomarkers and predict patients' likelihood of responding to ICBs.

RESULTS

Multiple views of the tumor microenvironment

Using bulk RNA-seq data combined with different types of biological prior knowledge, we derived five types of system-based signatures of the TME for 7,550 cancer patients across 18 solid cancers from TCGA data as summarized in Figure 1A (additional details in the experimental procedures).

The first type of signature consists of immune-cell fractions obtained with quanTIseq.[25] quanTIseq cell fractions are estimated using a deconvolution approach leveraging as prior knowledge cell-type-specific expression signatures for B cells, classically (M1) and alternatively (M2) activated macrophages, monocytes, neutrophils, natural killer (NK) cells, non-regulatory $CD4^+$ T cells, $CD8^+$ T cells, regulatory T (T_{reg}) cells, and myeloid dendritic cells. quanTIseq also provides the fraction of "other" unclassified cells in the mixture, resulting in a total of 11 cellular features.

We considered two types of signatures describing intracellular networks, quantifying the activity of 14 signaling pathways and 118 TFs. Pathway activity was derived using PROGENy,[23,26] which uses as prior knowledge perturbation-response gene signatures extracted measuring downstream gene changes upon perturbations of a pathway. The activity of the pathways is computed as linear combinations of their signature genes. TF activity was computed using DoRothEA,[24] which assumes as prior knowledge the networks of TF-target interactions (regulons) and infers the activity of each TF from the expression of its target genes.

In addition, we extracted two types of signatures related to intercellular networks, quantifying 813 LR pairs and 169 CC pairs. To compute LR pairs we leveraged as prior knowledge 1,894 LR pairs annotated in Ramilowski et al.[14] We filtered for literature-supported pairs expressed in 25 cell types that are present in the TME, including immune cells, cancer cells, fibroblasts, endothelial cells, and adipocytes (experimental procedures). The weight for the LR pairs was computed as the minimum of the expression of the ligand and the receptor.[27] We then computed a score of the CC interactions between 13 aggregated cell types (including autocrine signaling), as a weighted sum of the number of LR pairs expressed for each CC pair (experimental procedures).

For the same TCGA samples, we also computed 14 different transcriptomics-based scores of immune response (Table S1). All these scores were recently published and have been proposed as predictors of response to ICB therapy. We computed cancer-specific correlations between the 14 scores (Figure S1) and identified a subset of 10 scores that were highly correlated

CellPress
OPEN ACCESS

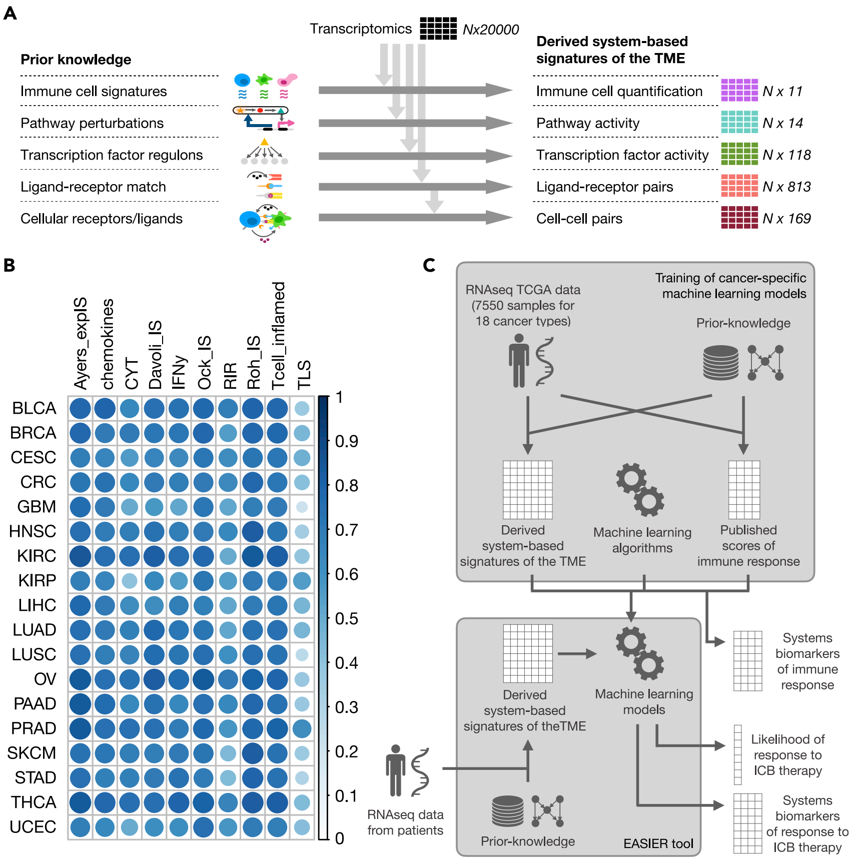

Figure 1. Overall description of the approach taken

(A) Derivation of the five system-based signatures of the TME based on the integration of RNA-seq data and different sources of prior knowledge.

(B) Cancer-specific median correlation of each of the 10 scores (described in Table S1) of immune response with all 14 other scores.

(C) Schematic pipelines. Cancer-specific models are trained on TCGA data. System-based signatures of the TME and scores of immune response are derived by combining RNA-seq data and prior knowledge, and are used respectively as algorithm inputs and outputs. Trained models are used to define system biomarkers of immune response. These models are also included in a tool called EaSIeR that allows users to input RNA-seq data for new patients and to compute the likelihood of response to immunotherapy and the biomarkers distinguishing responders and non-responders.

across all 18 cancer types (across cancer types median of the median Pearson correlation with all other scores >0.4, Figure 1B).

We considered these scores as output variables (*tasks*) and trained two different multi-task machine learning algorithms using the derived system-based signatures as input features (Figure 1C). Multi-task learning allows solving of multiple learning tasks at the same time, exploiting the shared information between tasks. Therefore, only the 10 correlated scores of immune response were used as tasks for model training. The first approach that we used is regularized multi-task linear regression (RMTLR)[28] using elastic net regularization (experimental procedures). Regularization allows one to improve model generalization (avoiding overfitting on the training data) and to perform selection of relevant predictive features (common for all tasks), which we interpreted as systems biomarkers of the immune response. The second approach that we used is Bayesian efficient multiple-kernel learning (BEMKL),[29] which was the best-performing algorithm in the NCI/DREAM7 challenge on prediction of cell line drug sensitivity from genomic information.[30] While BEMKL is a more sophisticated approach that can account for non-linearities, it does not allow us to directly select the important predictive features. Cancer-specific models were trained using RMTLR and BEMKL with randomized cross-validation using as input data each of the five system-based signatures (single views) separately, pairwise combinations, and the combination of all views (cross-validation performances in Figure S2). For RMTLR, the randomized cross-validation was also used to select only robust features (experimental procedures). The trained cancer-specific models are provided as a tool called EaSIeR (experimental procedures). Users can provide RNA-seq data and use the tool to derive system-based signatures of the TME, analyze systems biomarkers of immune response, and predict patient-specific likelihood of response to ICB therapy (Figure 1C).

For models optimized using RMTLR, we computed the median across cancer types of the estimated feature weights and verified that 99% of the feature weights (estimated separately for each view) had a variance ≤ 0.0015 across tasks, proving that biomarkers are in general consistent across tasks (Figure S3). By clustering tasks based on feature weights, we obtained four main clusters: (1) cytolytic activity (CYT),[31] (2) tertiary lymphoid structures (TLS) signature,[32] (3) chemokines[33] and interferon-γ (IFN-γ)[34] signatures (both related to cytokine production), and (4) all six remaining immune signatures (Figure S3).

We analyzed the systems biomarkers that we identified using RMTLR to predict estimated immune response, based on the different tasks, separately for each type of system-based signature of the TME, i.e., immune cells, intracellular networks (pathways and TF activity), and intercellular networks (LR and CC pairs). Then we assessed the performance of our models in predicting response to ICB therapy on independent datasets, analyzed systems biomarkers that differentiate responders and non-responders to therapy, and evaluated the effects of combining different types of signatures. The results of these analyses are presented in the following sections.

Immune cells as biomarkers of immune response

We identified several relevant robust associations between immune-cell composition and scores of immune response (Figure 2; experimental procedures). In particular, $CD8^+$ T cells, which are essential for tumor-cell recognition and killing,[35] were identified

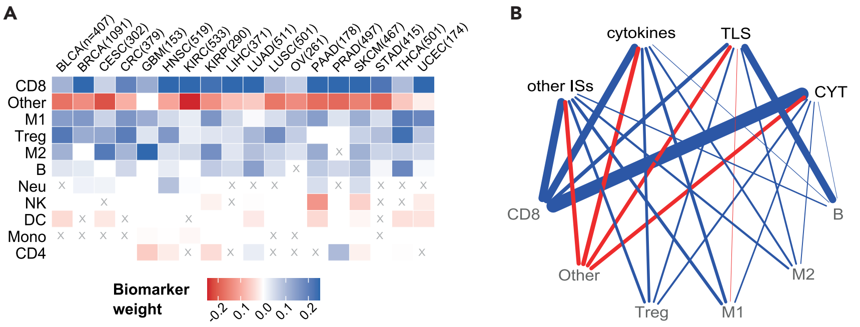

Figure 2. Systems biomarkers based on immune cell quantification

(A) Heatmap showing regression coefficients for cancer-type-specific models when using immune cell quantification as biomarkers. Shown are the median values computed first across 100 randomized cross-validation runs (to keep only robust biomarkers) and then across tasks. Biomarkers that are significantly different from zero (Wilcoxon rank-sum test, $p < 0.05$) in fewer than half of the tasks are marked with an X. Rows (biomarkers) were sorted according to their absolute mean value across tumors.

(B) Network representing associations between clusters of tasks (top nodes) and immune cell biomarkers (bottom nodes). Only the top five biomarkers for each cluster of tasks (ranked by median weight across the tasks in the cluster) that are significantly different from zero (Wilcoxon rank-sum test, $p < 0.05$) in at least half of the tasks of the cluster for at least half of the cancer types are shown. Edge widths represent the median weight of each biomarker across cancer types. Positive (blue), no (white), or negative (red) association of each biomarker with the tasks that are hallmarks of the immune response is depicted. B, B cells; CD4, non-regulatory $CD4^+$ T cells; CD8, $CD8^+$ T cells; DC, dendritic cells; IS, immune signature; M1, classically activated macrophages; M2, alternatively activated macrophages; Mono, monocytes; Neu, neutrophils; NK, natural killer cells; Treg, regulatory T cells.

as positive biomarkers for all cancer types (Figure 2A). The fraction of "other" uncharacterized cells positively correlates with tumor purity and negatively correlates with the percentage of tumor-infiltrating immune cells.[25] Here, we observed a negative correlation of this feature with immune response across all cancers, which can be interpreted as a positive association between immune infiltration levels and immune response (Figure 2A). Some immune cells, such as T_{reg} cells, M1 and M2 macrophages, and B cells, were positively associated with response in most cancer types (16, 17, 16, and 14, respectively, of the 18 analyzed cancer types; Figure 2A). The strong positive association of T_{reg} cells and M2 macrophages, which are immunoinhibitory immune cells, either might be due to a general association with a high immune-cell infiltration or might reflect negative-feedback mechanisms that arise to keep the immune system in check. For most of the cell types the association was consistent across all four clusters of tasks (Figure 2B), with the exception of B cells, $CD8^+$ T cells, and M1 macrophages. As expected, B cells showed a particularly strong association with the TLS signature. TLS are organized lymphoid aggregates, and recent studies suggested that B cells are localized in TLS and that B cells and TLS contribute to an effective T cell response to ICBs.[32,36] Instead, $CD8^+$ T cells and M1 macrophages are respectively less strongly or mildly negatively associated with TLS. As expected, $CD8^+$ T cells are positively associated with CYT, which is based on genes upregulated upon activation of $CD8^+$ T cells,[31] and with cytokines, some of which (e.g., IFN-γ) are expressed by active $CD8^+$ T cells.[33,34]

Intracellular networks as biomarkers of immune response

Tumor-cell intrinsic deregulation of cellular signaling due to mutations or epigenetic alterations has an effect on the functioning of intracellular networks that regulate cellular phenotype but also on the interaction with the immune system.[9] Among the analyzed pathways and TF activities we identified several biomarkers of immune response (Figure 3).

We identified a strong positive association between the TRAIL apoptotic pathway, the JAK-STAT pathway, and the NF-κB pathway and the predicted immune responses in all cancer types (Figure 3A; experimental procedures). The TRAIL pathway can be activated by different types of immune cells causing tumor cell apoptosis.[37] In turn, tumor cell death results in the activation of the immune system via the cancer-immunity cycle.[35] The JAK-STAT and NF-κB pathways are also known to play pivotal roles in immune responses. Both pathways are stimulated by IFN-γ released mainly by NK and T cells[38] and regulate several mechanisms of adaptive immune resistance, including upregulation of immune-checkpoint molecules,[10] inhibition of production of pro-inflammatory chemokines,[39] and promoting expression of class I MHC molecule expression.[13] Both JAK-STAT and NF-κB pathways showed a lower association with TLS than with other tasks (Figure 3B), suggesting that their role in the immune response is not dependent on TLS.

Activation of the PI3K pathway, which we identified as a biomarker for 16 cancer types (Figure 3A), was also shown to enhance PD-L1 expression.[40,41] PI3K pathway activation can be caused by different mechanisms of innate resistance to immune response, such as loss of *PTEN*, which is an inhibitor of the PI3K pathway, or oncogenic mutation of the *PIK3CA* gene. Direct or indirect therapeutic inhibition of PI3K was shown to reduce PD-L1 expression and increase antitumor immunity.[10]

The positive biomarkers described above are pathways generally associated with inflamed tumors, which are usually more predisposed to responding to ICB immunotherapy.[42] In contrast, for VEGF, we observed a negative association with immune response for 17 cancer types, and no association for ovarian cancer (OV) (Figure 3A). The negative association is in line with the role that VEGF plays in promoting immune exclusion due to the presence of vascular barriers.[42] Immune-excluded tumors are less responsive to ICB therapy,[43] and inhibition of VEGF could promote immune infiltration and improve efficacy when used in combination with ICB therapy.[44]

Similarly, the p53, hypoxia, and EGFR pathways also showed negative correlation for the majority of the cancer types (14, 13, and 9, respectively, Figure 3A). Interestingly, it has been recently shown that, in lung cancer, loss-of-function mutations in the tumor suppressor P53 gene are associated with increased expression of PD-L1, immune-cell infiltration, and tumor immunogenicity and may help in predicting response to ICB therapy.[45] Our findings suggest that the activity of the p53-mediated DNA damage response pathway might be considered as a predictor

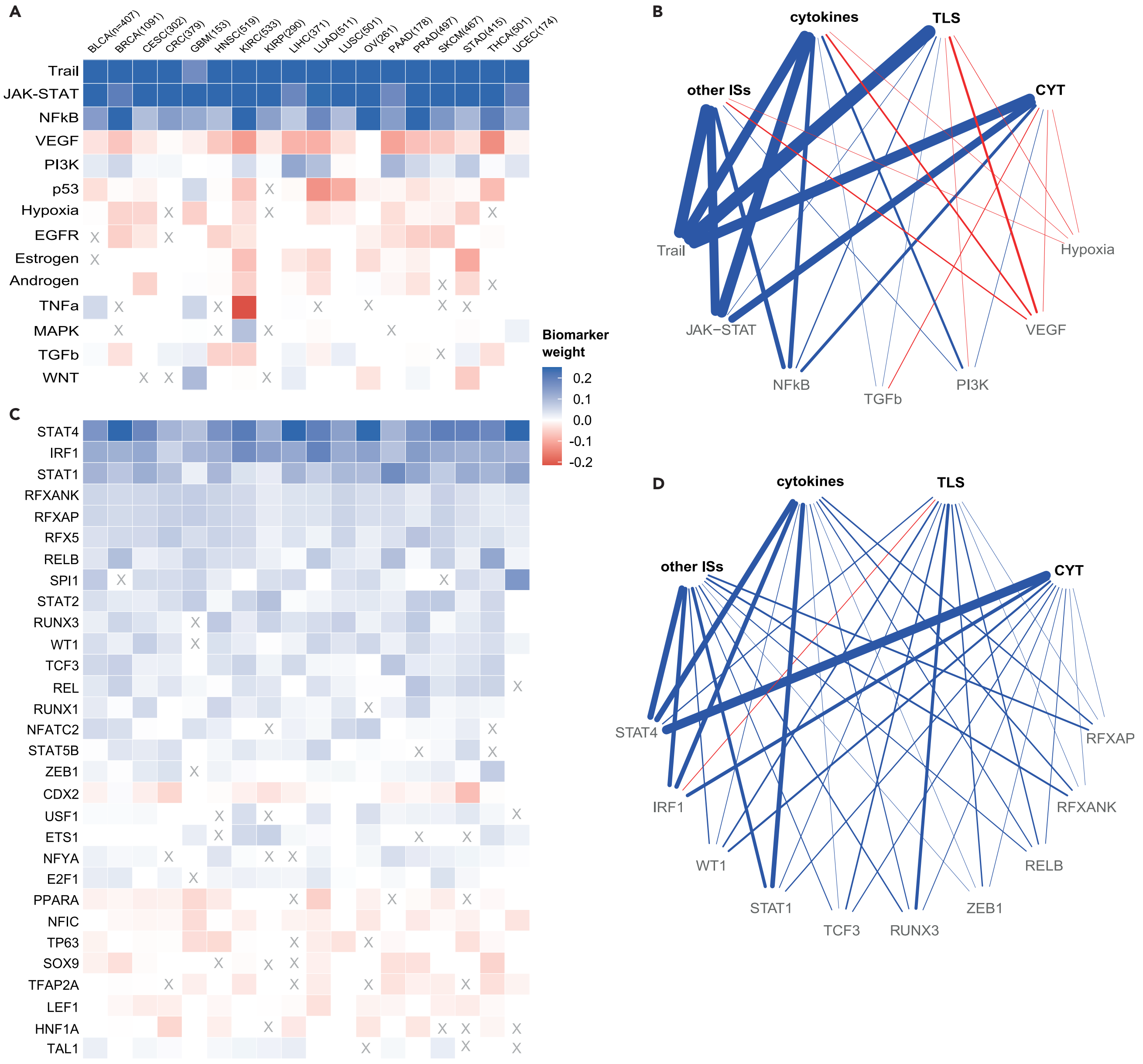

Figure 3. Systems biomarkers based on pathway and TF activity

Heatmaps showing regression coefficients for cancer-type-specific models when using (A) pathway activity and (C) TF activity (limited to the top 30) as biomarkers. Shown are the median values computed first across 100 randomized cross-validation runs (to keep only robust biomarkers) and then across tasks. Biomarkers that are significantly different from zero (Wilcoxon rank-sum test, $p < 0.05$) in fewer than half of the tasks are marked with an X. Rows (biomarkers) were sorted according to their absolute mean value across tumors. Networks representing associations between clusters of tasks (top nodes) and biomarkers (bottom nodes) from (B) pathway and (D) TF activity. Only the top five biomarkers for each cluster of tasks (ranked by median weight across the tasks in the cluster) that are significantly different from zero (Wilcoxon rank-sum test, $p < 0.05$) in at least half of the tasks of the cluster for at least half of the cancer types are shown. Edge widths represent the median weight of each biomarker across cancer types. Positive (blue), no (white), or negative (red) relationship of each biomarker with the tasks that are hallmarks of the immune response is shown.

of ICB therapy response as well, not only for lung cancer (LUAD and LUSC have the strongest associations), but also for other cancer types (Figure 3A). Notably, the p53 pathway revealed a positive correlation in glioblastoma multiforme (GBM); this is in agreement with stronger immune responses found in *TP53* wild-type GBM patients compared with patients harboring *TP53* mutations.[46]

We also observed a negative impact of hypoxia on the immune response (Figure 3A), consistent with recent observations that hypoxia impairs antitumor immunity and contributes to resistance to immunotherapy.[47] Preliminary studies in mice revealed the potential of targeting hypoxia in combination with ICB therapy to restore T cell infiltration and increase efficacy of immunotherapy.[48]

Consistent with our results (Figure 3A), it has been shown that activation of the EGFR signaling pathway contributes to an uninflamed TME, and that combining *EGFR* inhibitors and anti-PD-1/PD-L1 antibodies could improve therapeutic outcome in *EGFR*-mutant tumors.[49]

Next, we focused our analysis on the association between TF activity and immune responses (Figure 3C for the top 30 biomarkers, full list in Table S2). We identified several TFs that were selected consistently across the majority of cancer types. For instance, *STAT1*, *STAT2*, and *STAT4*, all selected as positive biomarkers (Figure 3C), are members of the STAT family in the JAK-STAT signaling pathway discussed above.[50] Although *STAT3* is often considered an important player in cancer immunotherapy,[51] it was not selected as top biomarker in our analysis. This is in line with recent publications suggesting that the main role in the regulation of PD-L1 expression is played by *STAT1*.[52] In tumor cell lines from several cancer types, small interfering RNA (siRNA) knockdown of *STAT3* did not reduce IFN-γ- or interleukin-27-induced PD-L1 protein expression, while siRNA knockdown of *STAT1* did.[53] *STAT4* deficiency has been associated with diminished antitumor immune response and worse prognosis.[54,55] The positive association of *STAT4* found in all 18 cancer types we analyzed suggests that this mechanism might play a major role in anticancer immunity and, possibly, response to ICB pan-cancer. As previously observed for the JAK-STAT pathway, the STAT TFs also seem to be not dependent on TLS (Figure 3D).

Another relevant biomarker (selected for all cancer types, Figure 3C) is *IRF1*. The *IRF1* TF can regulate the expression of PD-L1[50,56] and the production of cytokines, including the *CXCL9* chemokine that is responsible for recruiting antitumor immune cells.[39] Similarly, *RELB* (selected for 17 of 18 cancer types), which is part of the NF-κB complex, also regulates PD-L1 expression and inflammation.[57] *RELB* also regulates MHC-I gene transcription.[13] Tumor-immune infiltration favored by pro-inflammatory cytokines, susceptibility of cancer cells to immune-effector mechanisms such as MHC-I gene expression, and expression of PD-L1 are all hallmarks of effective immunotherapy.[58]

Other important positive biomarkers shared across all 18 cancer types were *RFXANK*, *RFXAP*, and *RFX5*, which form the RFX trimeric complex (Figure 3C). This complex cooperates with *NLRC5* to drive the transcription of class I MHC genes.[59] Accordingly, recent studies suggested that reduced activity of *NLRC5* plays a key role in immune evasion.[60,61] Taken together, these results may hint at RFX as a candidate biomarker of antitumor immunity.

Another positive biomarker was *RUNX3* (selected for 16 cancer types, Figure 3C), which plays a role in the TME regulating hypoxia and immune-cell infiltration, and has been suggested as a potential target to prevent immune escape of cancer cells.[62] We observed that *RUNX3* was more strongly associated with TLS than with other immune signatures (Figure 3D).

In addition, we found a number of biomarkers negatively associated with immune response, although the weight of the association was in general lower. *CDX2* was identified as a negative biomarker in 14 cancer types (Figure 3C). Loss of *CDX2* has been reported in colorectal (CRC) tumors that are microsatellite unstable[63] or PD-L1 positive.[64] These observations are in agreement with the negative association that we identified and suggest a potential role of *CDX2* as a biomarker of ICB therapy. Other results suggested that *CDX2* might also play an important role in other cancer types and in particular in stomach cancer (STAD). In our results, CRC and STAD showed the strongest negative associations with immune response for *CDX2*, but a negative association was identified also for other cancer types (Figure 3C). Another interesting example of a negative biomarker (for 11 of the 18 cancer types, Figure 3C) was *PPARA*, which encodes a ligand-activated TF regulating lipid metabolism and fatty acid oxidation.[65] The *PPARA* antagonist TPST-1120 promotes a more inflamed TME in different types of cancer and is currently in clinical trial as a monotherapy and in combination with anti-PD-1 therapy.[66] Blocking *PPARA* shifts the metabolic balance of immune cells from fatty acid oxidation toward glycolysis, which works in favor of certain immune-cell populations such as M1 macrophages and effector T cells, but against M2 macrophages and T_{reg} cells.[66] This was in line with the negative association that we observed for *PPARA*, because an inflamed microenvironment is essential for effective ICB therapy.

To compare the predictive powers of different approaches for quantifying intracellular pathways, we used cross-validation on TCGA data to assess the predictions of the scores of immune response obtained using the pathway activity scores and proteomics profiling (expression of 200 proteins and 58 phosphoproteins) using reverse-phase protein array (RPPA) data from The Cancer Proteome Atlas (Figure S4; experimental procedures).[67] Interestingly, the pathway activity scores derived with PROGENy from RNA-seq data revealed higher ability to predict scores of immune response for all cancer types, except UCEC ($p = 0.35$).

Biomarkers of immune response based on cell-cell communication

The phenotype of cancer cells is defined not only by intracellular oncogenic pathways but also by their exchange of signals with other TME cells. We, therefore, analyzed the potential of intercellular data in driving an effective immunotherapy response through LR and CC interactions among cell types of the TME (Figure 4; experimental procedures).

Among the LR (sender → receiver molecule) biomarkers (top 30 biomarkers in Figure 4A, full list in Table S3), we found several pairs of chemokines and corresponding receptors that are important for attracting immune cells to the TME.[12] An important chemokine in the TME is *CXCL10*, which regulates immune-cell migration, differentiation, and activation.[11] Higher levels of *CXCL10* are associated with increased number of infiltrated $CD8^+$ T cells.[12] The most studied receptor of *CXCL10* is *CXCR3* (LR pair associated with immune response in 12 of the 18 cancer types, Figure 4A), which is expressed by effector $CD8^+$ T cells, T helper 1 cells, and NK cells, which are all antitumor lymphocytes.[12] However, the top *CXCL10*-related LR pair, which we identified as a positive biomarker for all cancer types, is *CXCL10* → *SDC4* (Figure 4A). While there is limited evidence on the role of *SDC4* in cancer, it has been shown that interaction between *CXCL10* and *SDC4* inhibits fibroblast recruitment in pulmonary fibrosis.[68] Our results suggest that a similar mechanism might take place in the tumor, since fibroblasts recruited in the TME can suppress the immune response

CellPress
OPEN ACCESS

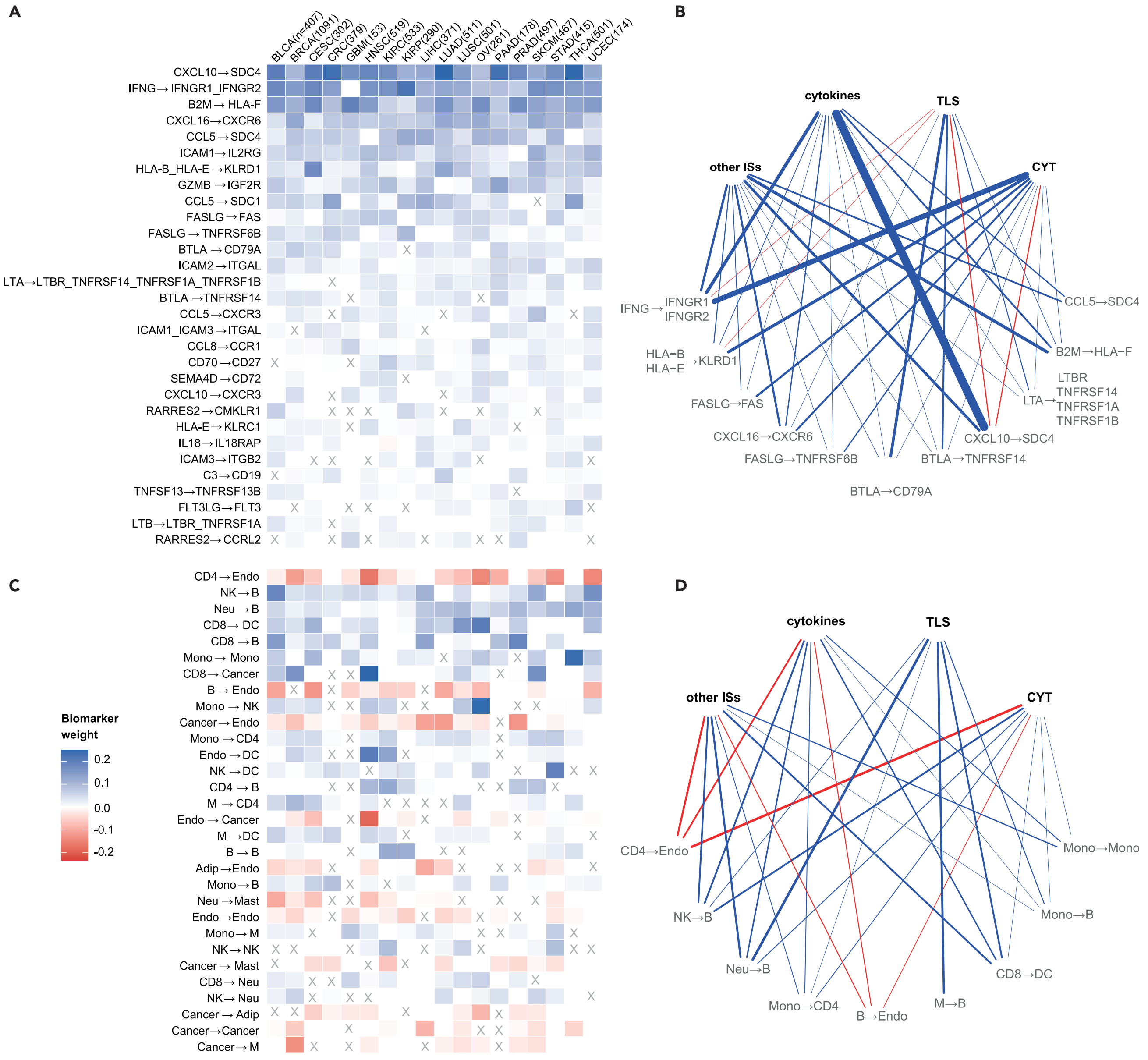

Figure 4. Systems biomarkers based on cell-cell interactions

Heatmaps showing top 30 regression coefficients for cancer-type-specific models when using (A) ligand-receptors pairs and (C) cell-cell pairs as biomarkers. Shown are the median values computed first across 100 randomized cross-validation runs (to keep only robust biomarkers) and then across tasks. Biomarkers that are significantly different from zero (Wilcoxon rank-sum test, $p < 0.05$) in fewer than half of the tasks are marked with an X. Rows (biomarkers) were sorted according to their absolute mean value across tumors. Networks representing associations between clusters of tasks (top nodes) and biomarkers (bottom nodes) from (B) ligand-receptor and (D) cell-cell pairs. Only the top five biomarkers for each cluster of tasks (ranked by median weight across the tasks in the cluster) that are significantly different from zero (Wilcoxon rank-sum test, $p < 0.05$) in at least half of the tasks of the cluster for at least half of the cancer types are shown. Edge widths represent the median weight of each biomarker across cancer types. Positive (blue), no (white), or negative (red) relationship of each biomarker with the tasks that are hallmarks of the immune response is shown. Arrows in biomarker names indicate the direction of the interaction (including cases of autocrine signaling).

and limit ICB immunotherapy efficacy.[69] In agreement with the role of *CXCL10*, the *CXCL10* → *SDC4* pair was particularly strongly positively associated with cytokines related to immune signatures, while showing a small negative association with TLS and CYT (Figure 4B).

CCL5 is another chemokine that we identified as positively associated with the immune response when bound to different receptors (*SDC4*, *SDC1*, and *CXCR3* for 16, 15, and 13 cancer types, respectively, Figure 4A). *CCL5* binding to *CCR5* is often described as a main actor of tumor progression.[70] However, recent studies have also shown that *CCL5* overexpression can favor $CD8^+$ T cell infiltration, antitumor immunity, and immunotherapy response,[71] in agreement with the positive association that we identified. Similarly, we found the *CXCL16* →

CXCR6 LR pair to be positively associated with the immune response for all 18 cancer types. Although the role of *CXCL16* in cancer is disputed,[72] overexpression of *CXCL16* by tumor cells is associated with increased infiltration of CD8$^+$ T cells[73] and NK cells.[74]

Another relevant LR biomarker was *IFNG* binding to *IFNGR1* and *IFNGR2* (positive association for 17 of the 18 cancer types, Figure 4A). *IFNG* is the gene encoding the IFN-γ cytokine, which, as already discussed in the pathways section, plays a main role in the immune response.[75] Mutations in the IFN-γ pathway (including *IFNGR1* and *IFNGR2*) are associated with resistance to ICB therapy.[76] Also, in this case, we observed that IFN-γ-dependent mechanisms of the immune response are independent of TLS (Figure 4B).

We also observed several positive biomarkers related to T-cell-mediated cancer cell killing (Figure 4A). These include the *GZMB* gene, which encodes the granzyme B serine protease that is secreted by CD8$^+$ T cells and NK cells and induces apoptosis in target cells binding to the corresponding receptor *IGF2R*.[77] In line with our results, upregulation of the receptor on the membrane of tumor cells promotes penetration of granzyme B, favoring immune-cell-mediated apoptosis, and was suggested as a potential target for immunotherapy.[78] Similarly, Fas ligand (encoded by *FASLG* gene) binding to the *TNFRSF6/FAS* receptor (part of the TNF receptor superfamily) is involved in CD8$^+$ T-cell- and NK-cell-mediated apoptosis.[79] Another interesting apoptosis-related biomarker is *BTLA*, which is a ligand for *CD79A* and *TNFRSF14*. Binding of *BTLA* to *TNFRSF14* (also known as *HVEM*) is an immune checkpoint that has been generally associated with negative immune responses,[80] although there is also evidence that this binding promotes survival of CD8$^+$ T cells in melanoma.[81] Another member of the TNFR superfamily that we identified as a biomarker is CD27, which is expressed by CD8$^+$ T cells and binds to its receptor *CD70* on antigen-presenting cells upon T cell activation. The *CD27/CD70* axis can be targeted with antibodies and its blockade has potential for cancer immunotherapy.[82]

We additionally identified as positive biomarkers several interactions between MHC-I genes and the corresponding receptors (*HLA-B_HLA-E* → *KLRD1* and *HLA-E* → *KLRC1* in 17 and 14 cancer types, respectively). These receptor genes encode *CD94/NKG2*, which is a family of inhibitory receptors expressed mainly on the surface of NK and CD8$^+$ T cells.[83] Anti-NKG2A antibody is a checkpoint inhibitor in clinical trials that was reported to enhance tumor immunity by promoting functioning of these immune cells.[83] Another relevant positive biomarker for all 18 cancer types is *B2M* → *HLA-F*. Although this is an intracellular interaction rather than an LR pair, the importance of this interaction for the immune response is clear, since *B2M* stabilizes the MHC-I complex, allowing recognition by the T cell receptor.[84]

Another important mechanism that we identified among the LR biomarkers is the stimulation of LFA-1 (encoded by *ITGAL* and *ITGB2* genes) by ICAM (*ICAM2* → *ITGAL*, *ICAM1_ICAM3* → *ITGAL*, and *ICAM3* → *ITGB2* in 18, 11, and 8 cancer types, respectively). LFA-1 is essential for the adhesion of CD8$^+$ T cells and NK cells to the cancer cell, thereby allowing their activation.[85] On mouse models, the ICAM1-LFA-1 interaction was also shown to cause clusters of activated T cells in the tumor and was suggested as a mechanism of tumor-mediated immune retention that prevents trafficking of T cells to lymph nodes.[86] In the same paper they suggest *ICAM-1* as a potential target for cancer treatment by incrementing lymphocyte migration to the lymph nodes.

We went further and analyzed the number of active LR interactions per CC pair, generating a score for each CC (sender → receiver cell) pair. CC scores were used to build a model that allowed us to disentangle the complex cross talk between cells of the TME and its influence on immune responses (top 30 biomarkers in Figure 4C, full list in Table S4).

CC communication profiles were very specific for each cancer type, with no biomarker shared across all cancer types (Figure 4C). The sign of the association with immune response, however, tended to be consistent across the different cancer types. Endothelial cells appeared as receiver cells for 5 of the top 30 CC pair biomarkers (with CD4$^+$ T cells, B cells, cancer cells, adipocytes, and endothelial cells as sender cells). In all cases they had a consistent, negative association for all cancer types for which they were selected as biomarkers. The weight of this association was consistent for all tasks except TLS, which showed no association (Figure 4D). Endothelial cells contributed to establishing an immunosuppressive TME, being actively involved in immune-cell exclusion and inhibition of lymphocyte activation.[87] The negative association that we identified is in line with the fact that inhibition of endothelial cells favors an antitumor immune response.[87] As expected, signaling of CD8$^+$ T cells to dendritic cells and cancer cells was identified as a strong positive biomarker (in 14 and 10 of the 18 cancer types, respectively, Figure 4C), in line with the crucial role these cells play in the immune response and mediation of immunotherapy effects.[88] Among the top 30 biomarkers, we also found B cells as receiver cells in 6 different CC pairs (with NK cells, neutrophils, CD8$^+$ T cells, CD4$^+$ T cells, and B cells as sender cells and monocytes). In all cases, these CC pairs were positively associated with the immune response, which is in line with the role of B cells as popular factories of antibodies after antigen recognition. In particular, NK cells and neutrophils help B cells by regulating their activation (NK cells → B cells and neutrophils → B cells were selected in 15 and 16 cancer types; Figure 4C).[89,90]

Prediction of response to immunotherapy targeting PD-1/PD-L1 immune checkpoints

We next assessed the performance of EaSIeR on seven different independent datasets, including four different cancer types, with patients treated with anti-PD-1 or anti-PD-L1 immunotherapy (experimental procedures; Table S5).[91–97] Pre-therapy RNA-seq data were provided as input to EaSIeR, which derived patent-specific, system-based signatures of the TME. The cancer-type-specific machine learning models, built on TCGA data to predict scores of immune response, were used to predict patient likelihood of response to ICB therapy (experimental procedures). We verified the overall advantage of using cancer-specific models by comparing predictive performance of the cancer-specific model for each dataset with respect to the models built for the remaining 17 cancer types (Figure S5; experimental procedures).

First, we assessed model performance in stratifying patients into responders and non-responders on two melanoma datasets

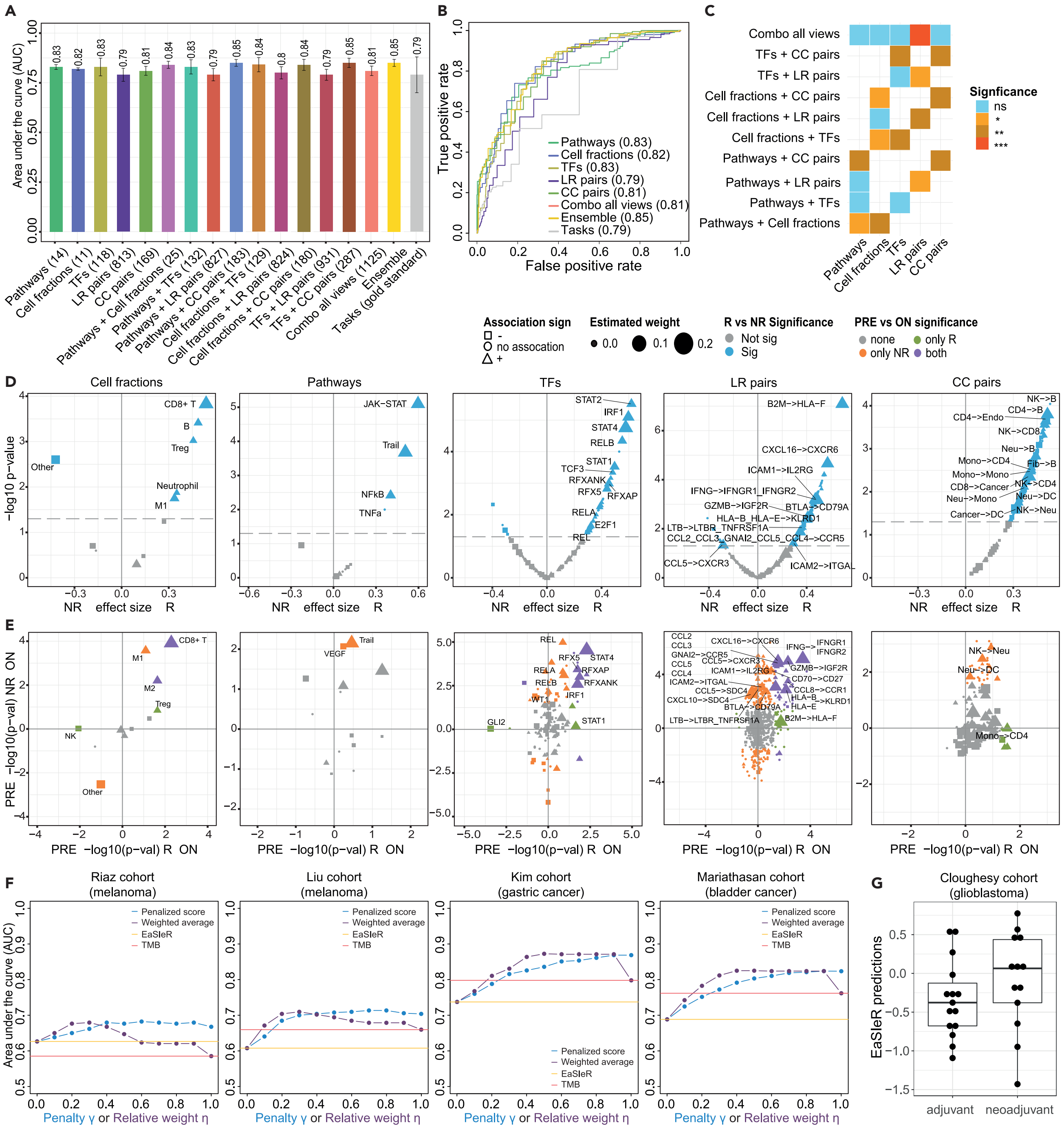

Figure 5. EaSIeR evaluation of independent datasets of patients treated with anti-PD-1/anti-PD-L1 immunotherapy

(A) Area under the curve (AUC) values for the Auslander and Gide melanoma cohorts[91,92] of predictions obtained using EaSIeR based on system-based signatures of the TME considering single views, pairwise combinations of views, combination of all views, average of single-view predictions (ensemble), and the computed tasks (gold standard). Bar plots represent the average AUC across tasks and error bars describe the corresponding standard deviation.

(B) Corresponding receiver operating characteristic (ROC) curve for the Auslander and Gide melanoma cohorts based on system-based signatures of the TME (single views), combination of all views, average of single-view predictions (ensemble), and the computed tasks (gold standard). ROC curves were computed as the average of the ROC curves obtained for each task.

(C) Performance comparison between single (x axis) and combined (y axis) views for the Auslander and Gide melanoma cohorts (one-sided Wilcoxon signed-rank test). Statistical significance is indicated by colors according to the legend. The significance level (*p < 0.05, **p < 0.01, ***p < 0.001) indicates whether combining views improves the performance.

(D) Volcano plots for systems biomarkers of the immune response from the Auslander and Gide melanoma cohorts comparing non-responder (NR) and responder (R) patients (two-sided Wilcoxon rank-sum test). Significant biomarkers (p < 0.05) are shown in blue. Biomarkers are drawn according to their corresponding sign

(legend continued on next page)

(Gide and Auslander cohorts) (Figures 5A–5C using RMTLR, results for BEMKL in Figure S6). For this, we used models built separately on each of the five described system-based signatures of the TME (single views), pairwise combinations of views, and combination of all views. RMTLR applied to the single views was able to accurately predict patient response (average area under the curve [AUC] = 0.79–0.84), with performance comparable or superior to the gold standard, i.e., the different tasks (average AUC = 0.79; Figures 5A and 5B). In particular, the ensemble model (average AUC = 0.85), computed as the average of the predictions from the single views, performed significantly better than the average of the literature-based tasks ($p = 0.003$, effect size = 0.849). Combining pairs of different views significantly improved performance, in particular for cell fractions + CC pairs, pathways + cell fractions, pathways + CC pairs, TF + CC pairs, and cell fractions + TF for RMTLR (Figure 5C; experimental procedures). Particularly good predictions were obtained combining information on pathways and cell fractions (average AUC using RMTLR = 0.84), despite the very limited number of features used (25 in total), performing even better than the combination of all views (average AUC using RMTLR = 0.81).

Unlike previous predictors that are based on simple gene sets, the EaSIeR systems-biology approach allows investigation of the mechanisms behind the differential patient responses to treatment. For the Gide and Auslander melanoma cohorts, we investigated how the identified systems biomarkers differ between responding and non-responding patients and evolve upon treatment (comparing pre- and on-treatment data). As expected, the top biomarkers for all individual views were the best at discriminating between responders and non-responders (Figure 5D). Responders had a higher number of infiltrated immune cells, including $CD8^+$ T cells, and a lower number of "other" non-immune cells. They also had higher activity in pathways (e.g., JAK-STAT, NF-κB) and TFs (e.g., *STAT1/2/4*, *IRF1*, *RELB*) that are upregulated in response to IFN-γ released by $CD8^+$ T cells during the immune response. Responders also showed more active CC interactions. Overall, these observations suggest that responders had a more active immune response in the tumor even before treatment with immunotherapy. Interestingly, applying the model to on-treatment data showed a significant increase in performance with respect to the pre-treatment point, especially for models based on pathways and cell fractions (Figure S7). To investigate this improvement, we compared the distribution of the systems biomarkers pre- and on-treatment in responders and non-responders (Figure 5E). As expected, after treatment, top positive biomarkers generally tended to increase in both responders and non-responders. This was the case, for example, for $CD8^+$ T cell abundance, *STAT1/2/4* and *RFX*-associated TFs, and IFN-γ binding to the corresponding receptor. In contrast, the fraction of "other" (non-immune) cells decreases upon treatment, suggesting a reduction in tumor size. Overall, these observations are in agreement with an increased antitumor immunity upon treatment with immunotherapy.

Motivated by the rationale that immune response and tumor antigenicity or foreignness are complementary hallmarks of successful ICB therapy,[58] we explored the potential of combining these two scores for predicting patient response. For this purpose, we applied EaSIeR to four datasets where both RNA-seq data and information on tumor mutational burden (TMB), considered as a proxy of tumor immunogenicity, were available (Table S5, experimental procedures). These datasets consist of two melanoma cohorts (Riaz and Liu), one gastric cancer cohort (Kim), and one bladder cancer cohort (Mariathasan). The performance of EaSIeR predictions was variable between datasets (average AUC of the ensemble model = 0.63, 0.59, 0.68, and 0.65 for Riaz, Liu, Kim, and Mariathasan, respectively; Figure S8). We used two different approaches to integrate EaSIeR predictions and TMB, one consisting in adding a negative or positive penalty to patients with low or high TMB, respectively, and the other as a weighted average of the two scores (experimental procedures). For all datasets and cancer types, we observed that both EaSIeR and TMB predictions can be improved by combining these two sources of information (Figure 5F). These results support the notion that these two scores provide orthogonal information and that they are equally important for effective prediction of treatment outcome as highlighted by the fact that the performance of the weighted average is the higher for intermediate values of the relative weight.

Finally, we applied EaSIeR to a glioblastoma cohort (Cloughesy) to study the effect of neoadjuvant immunotherapy on immune response.[97] Glioblastoma is associated with poor survival after standard of care surgery (median progression-free and overall survival of 7 and 15 months, respectively).[97] The findings by Cloughesy and colleagues suggest that neoadjuvant administration of anti-PD-1, continued with adjuvant therapy after surgery, can extend overall survival with respect to administering only adjuvant therapy, due to an enhanced antitumor immune response. In line with this finding, EaSIeR predicted a stronger immune response for patients treated with neoadjuvant ICB therapy (Figure 5G). However, due to the limited size of the cohort (28 patients: 15 adjuvant, 13 neoadjuvant), these results should be interpreted carefully as they are not highly statistically significant

(shape) and weight (size) obtained during model training. Labels are reported for the top 15 cancer-specific biomarkers (based on the association with the tasks) that are significantly different between R and NR.

(E) Starburst plots showing the statistical comparison ($-\log_{10}$ p, two-sided Wilcoxon rank-sum test) between pre- and on-treatment samples for responders (R, x axis) and non-responders (NR, y axis). The sign is used to show if the biomarkers are higher ON (positive sign) or PRE (negative sign) treatment. Biomarkers are colored according to their consistent statistical significance in both NR and R patients. Labels are reported for the top 15 cancer-specific biomarkers (based on the association with the tasks) that are significantly different between R and NR.

(F) AUC values obtained combining EaSIeR predictions (ensemble model averaged across tasks) and information on tumor mutational burden (TMB) for two melanoma datasets (Riaz and Liu cohorts),[93,94] one gastric cancer dataset (Kim cohort),[96] and one bladder cancer dataset (Mariathasan cohort).[95] The x axis represents the penalty γ or the relative weight η, depending on the approach used to combine the two scores ("penalized score" or "weighted average," respectively). In both cases low values of the parameter give more importance to EaSIeR predictions, while high values give more importance to the TMB.

(G) Boxplot of EaSIeR predictions comparing predicted outcome in the case of adjuvant and neoadjuvant therapy in the glioblastoma dataset (Cloughesy cohort).[97]

CellPress
OPEN ACCESS

(Wilcoxon rank-sum test, p = 0.1, effect size = 0.23). We observe that patients who received neoadjuvant therapy have increased activity of the Trail pathway, possibly as a consequence of the more effective killing of tumor cells, and of the TFs IRF1, responsible for stimulating recruitment of antitumor immune cells,[39] and NFATC2, a marker of T cell activation, which could explain the stronger immune response of these patients (Figure S9).

DISCUSSION

The efficacy of immunotherapy with immune-checkpoint inhibitors depends on the intricate cross talk across the cells in the TME. Thus, to disentangle the mechanisms underlying—and ultimately predicting—patient response, it is essential to adopt a holistic strategy to study patients' tumors.[1,98] To find effective biomarkers, it is essential to use a systems biology approach to investigate how different mechanisms contribute to the overall behavior, looking at the TME from different perspectives.[6,99] Toward this goal, in this study, we derived systems biomarkers of immune response considering the cellular composition of the TME together with inter- and intracellular communication to provide a more comprehensive and mechanistic characterization of tumors. Since RNA-seq data are becoming routinely available in clinical settings, we decided to focus on deriving system-based signatures by using prior knowledge to structure RNA-seq data into different mechanistic layers. Importantly, our approach proved to be effective in predicting responses to ICB for independent datasets.

We used machine learning to look for associations between the derived system-based features and the immune response, estimated using 14 predictors (proxies) derived from recent publications. We considered these proxies as different tasks to be predicted by our machine learning models and used multi-task learning algorithms in order to learn all tasks jointly. Multi-task techniques have the great advantage that they allow one to exploit the shared information across tasks. By inducing regularization forcing the algorithms to perform well in all tasks, they prevent overfitting, thereby providing more generalizable models. Another advantage of our approach is that it does not require a dataset where patients' responses to immunotherapy are known for model training. These datasets are generally limited to a few dozen patients and are, therefore, not optimal for training of machine learning approaches due to the risk of overfitting. Instead, we could exploit the large sample size of the TCGA RNA-seq dataset to build cancer-specific models, including cancer types for which the potential of ICB therapy has not been extensively studied yet. An additional advantage of our approach is that by deriving system-based signatures, it performs knowledge-guided dimensionality reduction (e.g., pathways consist of only 14 features derived from thousands of genes). Unlike other approaches for dimensionality reduction, which cause loss of interpretability of the derived features, our derived signatures actually improve the interpretability, providing quantification of different complex mechanisms of the TME. By aggregating RNA-seq data into higher representations, our models provide views of the tumor that would have been accessible only through the generation of additional data with complex and expensive techniques (e.g., imaging for immune-cell quantification, phosphoproteomics upon perturbation for pathway activation). Dimensionality reduction also allows improving algorithm performance, reducing the risk of overfitting.

In summary, our approach allows us to derive more generalizable models thanks to: (1) multi-task learning, leveraging information from multiple proxies of the immune response; (2) training on large datasets from TCGA; and (3) dimensionality reduction using system-based features. This results in superior predictive power on completely independent datasets, outperforming also the gold standard based on the different literature-derived proxies of the immune response.

An intriguing observation is that intracellular signaling pathway activity is a major predictor of the immune response. This is likely due to the fact that they regulate both intrinsic (due to mutations) and extrinsic (due to exogenous stimulation) tumor cell mechanisms of resistance to immune attack. Importantly, in our analysis, we found that pathway activity was a better predictor than protein expression or phosphorylation. The limited predictive power of proteomics data might be partially due to the use of RPPA data, which are limited to a few hundreds of proteins and can be noisy. However, we believe that a main motivation for the superior performance of the pathway activity scores is that they were derived from perturbation-response signatures, therefore inferring the activity of the pathway by looking at how genes downstream of the pathways respond to perturbations.[23] This approach allows us to take into account post-translational modification and capture the dynamic nature of the pathways even when only static RNA-seq data are available. Similar observations hold for TF activity, which was computed based on the expression of the regulated genes.[100]

For both pathways and TFs, we identified biomarkers that could be used to suggest new therapeutic strategies. Intracellular networks regulate tumor cell interactions with the microenvironment via regulation of immune checkpoints, regulation of antigen presentation, and release of inflammatory chemokines.[9] Targeting these intracellular networks in cancer cells has the potential to improve the efficacy of immunotherapy with ICBs[101] or to be an alternative approach to immunotherapy by inhibiting the expression of immune checkpoints.[10,102] For example, overactivation of the PI3K pathway, which we identified as a positive biomarker, was shown to cause overexpression of PD-L1, contributing to immune evasion.[103] PI3K inhibition resulted in downregulation of PD-L1 in different cancer types, providing an alternative approach to ICBs to enhance antitumor immunity.[10] The VEGF pathway, identified instead as a negative biomarker of immune response in our analysis, has been already associated with immune exclusion and resistance to ICB therapy.[42] In line with our results, accumulating evidence suggests that combining ICB immunotherapy with antiangiogenic agents targeting VEGF might improve the clinical efficacy of immunotherapy in patients with lung cancer.[44] Similarly, the TF *PPARA*, identified as a negative biomarker in our analysis, could be inhibited to augment inflammation in the TME. In line with this, a recent clinical trial showed that *PPARA* blockade promotes a more inflamed TME and improves ICB efficacy in advanced solid cancers.[66] These results suggest that our biomarkers have strong potential to be exploited in future research to suggest new personalized therapies.

As expected, the tumor immune-cell composition was also important for predictions. However, complementing immune-

cell fractions with pathway information significantly improved the predictive power of both individual views. Both the pathway activities and the immune-cell fractions are derived using gene signatures, which might justify their high association with antitumor immune response, as shown by their superior prediction performance. As highlighted by our results, another important aspect of prediction of immune response is the intercellular communication. Although these types of communication are still less explored, our results suggest that they deserve more attention in future research. Our current approach to derive intercellular interaction signature relies on only prior mechanistic knowledge. In this regard, more refined approaches to infer CC networks from bulk RNA-seq data, e.g., by integrating gene expression analysis and the exploitation of orthogonal information from single-cell technologies, hold great potential.[104]

Remarkably, we found literature support validation for most of our top biomarkers. This highlights the potential of using a systematic and unbiased approach like the one described in this paper. A word of caution when interpreting the results is that the derived associations do not provide the direction of the causal effects. Therefore, as we observe, for both immune-cell quantification and pathway activity, that the identified biomarkers can be either the drivers or the result of the anticancer immune response.

Instead of focusing on individual mechanisms requiring specific biological assays, we use widely available RNA-seq data complemented by prior knowledge to provide a holistic picture of the TME. In this way, we provide a tool (EaSIeR) that can be readily used to predict individual patients' responses to ICB therapy and paves the way to suggesting new therapeutic strategies not only for individual tumor types but also for individual patients, based on systems biomarkers. We expect that different data modalities will become increasingly available in clinical practice and will provide complementary information on the TME that could be integrated into EaSIeR. In this paper we provide a proof of principle of how EaSIeR scores and TMB, which provide orthogonal information on antitumor immune responses, can be effectively integrated to more optimally predict the response to ICB therapy in patients with different cancer types. With the advancement of computational pathology, we envision that it will be possible to extract information on tumor composition and spatial localization of immune cells[105,106] to be integrated in EaSIeR to derive spatial biomarkers and possibly improve predictive power. Finally, emerging single-cell methodologies that allow us to look at the TME from a different angle can provide complementary insights into intra- and intercellular interactions[27,107,108] that could be used to adapt the EaSIeR framework to single-cell analyses.

EXPERIMENTAL PROCEDURES

Resource availability

Lead contact

Federica Eduati is the lead contact for this study and can be reached at f.eduati@tue.nl.

Materials availability

There are no newly generated materials associated with this paper.

Data and code availability

All the datasets used are publicly available (Table S5). The code used for model training and analysis is available at https://github.com/olapuentesantana/mechanistic_biomarkers_immuno-oncology, and the EaSIeR R package https://github.com/olapuentesantana/easier_manuscript to compute systems biomarkers and likelihood of patient response to ICB from RNA-seq data is available at.

TCGA RNA-sequencing data

Gene expression data for 18 solid tumors were downloaded via the Firehose tool from the BROAD Institute (https://gdac.broadinstitute.org), released January 28, 2016. We selected primary tumor or metastatic (only in the case of melanoma) samples, resulting in a total of 7,750 patients.

We extracted the gene expression data from "illuminahiseq_rnaseqv2-RSEM_genes" files. From these data, we used "raw_count" values as counts and we calculated transcripts per million (TPM) from "scaled_estimate" values multiplied by 1,000,000. We first removed those genes with a non-valid HGNC symbol and then we averaged the expression of those genes with identical HGNC symbols.

Validation data

Validation cohorts for melanoma (Gide, Auslander, Riaz, and Liu cohorts),[91–94] gastric cancer (Kim cohort),[96] bladder cancer (Mariathasan cohort),[95] and glioblastoma (Cloughesy cohort)[97] were derived from published datasets of patients treated with anti-PD-1/anti-PD-L1 therapy with publicly available RNA-seq data (Table S5 for more details and accession numbers).

For the Auslander, Gide, Riaz, Kim, and Cloughesy cohorts, we downloaded the corresponding SRA files from the Sequence Read Archive (SRA; https://www.ncbi.nlm.nih.gov/sra/) and converted to FASTQ using the "fastq-dump function" provided by the SRA toolkit. FASTQ files of RNA-seq reads were then pre-processed with quanTIseq to obtain gene counts, TPM, and cell fractions.[25] In brief, we used Trimmomatic[109] to remove adapter sequences and read ends with Phred quality scores lower than 20, discard reads shorter than 36 bp, and trim long reads to a maximum length of 50 bp (quanTIseq pre-processing module). We ran Kallisto[110] on the pre-processed RNA-seq reads to generate gene counts and TPM using the "hg19_M_rCRS" human reference (quanTIseq gene-expression quantification module). For the Mariathasan cohort, gene counts were obtained using the IMVigor 210 Biologies R package. Counts and TPM data for the Liu cohort were downloaded from the supplementary files of the study.

For the Riaz, Liu, Kim, and Mariathasan cohorts we also considered the available information on TMB as provided, already processed in the original publications. Whole-exome sequencing (WES) was used to quantify the TMB, except for the Mariathasan cohort, for which panel sequencing was used instead. For the Riaz, Liu, and Kim cohorts, the TMB was defined as the total number of non-synonymous mutations detected from WES, whereas, in the Mariathasan cohort, panel sequencing was used to estimate the TMB by including synonymous mutations in addition to non-synonymous mutations. The TMB was provided as mutations per megabase, except for the Kim cohort, for which the TMB was available as a categorical variable with three classes: low (<100), moderate (100–400), and high (>400).

For all datasets we considered only patients treated with anti-PD-L1 or anti-PD-1. For the Auslander cohort we considered the classification of responders and non-responders as in the original publication, as response evaluation criteria in solid tumors (RECIST) were not provided. For the Mariathasan cohort we considered patients with complete response as responders and patients with progressive disease as non-responders, in agreement with Bonavita et al.[111] For all the other datasets we considered responder patients to have complete response or partial response, and non-responder patients to have partial response or stable disease. Since RECIST classification was not provided for the glioblastoma Cloughesy cohort, we compared our model predictions with the type of therapy (neoadjuvant versus adjuvant) to verify whether we could predict the expected better therapeutic outcome for patients following neoadjuvant anti-PD-1 therapy.[97] More details can be found in Table S5.

System-based signatures of the TME

We used RNA-seq data to derive different types of mechanistic signatures integrating prior knowledge.

Immune-cell quantification

We used quanTIseq to compute tumor-infiltrating immune-cell fractions, which are estimated by applying deconvolution to bulk gene expression levels in a mixture based on cell-specific gene-expression signatures.[25]

quanTIseq returns the fractions of 10 cell types: B cells, classically (M1) and alternatively (M2) activated macrophages, monocytes, neutrophils, NK cells, non-regulatory CD4+ T cells, CD8+ T cells, T_{reg} cells, and myeloid dendritic cells. The fraction of other cell types in the mixture is computed as 1 minus the total fraction of immune cells and was shown to often correlate with tumor purity.[25] Since non-regulatory CD4+ T cells are difficult to distinguish from T_{reg} cells, we decided to consider non-regulatory CD4+ T cells as the sum of both non-regulatory CD4+ T cells and T_{reg} cells, keeping T_{reg} cells as a separate cell type as well.

Pathway activity

We used PROGENy to compute scores for 14 pathways: androgen, EGFR, estrogen, hypoxia, JAK-STAT, MAPK, NF-κB, p53, PI3K, TGF-β, TNF-α, Trail, VEGF, and WNT.[23,26] Pathway-specific signatures were derived from pathway-perturbation experiments by investigating which genes change in expression when a pathway is perturbed. A linear regression model was used to fit the genes that were affected by the perturbation of the pathway. Both pathway-specific signatures and gene expression data were then used to infer pathway signaling activity. The pathway scores were directly computed using the PROGENy R package version 1.10.0. Since these scores are a linear transformation of gene expression data, we removed 448 genes used to compute the proxies of immune response (average pan-cancer Pearson correlation with original pathway activity = 0.99, $p < 10^{-16}$).

Transcription factor activity

We used DoRothEA to compute TF activity.[100] The expression of individual genes is controlled by TFs, and TF activity can be estimated by the expression of its target genes (so-called TF regulons). The TF activity was estimated using analytic Rank-based Enrichment Analysis (aREA) from the Viper R package 1.22.0, as part of the DoRothEA R package 1.0.1. aREA provides a normalized enrichment score for each TF regulon based on the average ranks of its targets. Each TF-target interaction is assigned a degree of confidence (from A to E) depending on the total supporting evidence. To consider only high-quality regulons, we filtered for confidence levels A and B, resulting in a total of 115 TFs.

Ligand-receptor pairs

Based on LR pair annotations from the database by Ramilowski et al.[14], we quantified LR interactions in the TME for each individual patient. This was done in two steps: first we derived a subset of 867 LR pairs that are potentially present in the TME, then we quantified these pairs for each patient based on RNA-seq data.

For the first step we started from the 1,894 LR literature-supported pairs in the Ramilowski database, consisting of 642 unique ligands and their 589 cognate receptors. Furthermore, the database annotates the TPM expression of these ligands and receptors in 144 human cell types based on cap analysis of gene expression (CAGE) from the FANTOM5 expression atlas. We filtered for the 24 cell types commonly acknowledged to be present in the TME and present in the Ramilowski database (Table S6). In addition, we considered a pan-cancer cell type derived using data from the Cancer Cell Line Encyclopedia (CCLE).[112] In the CCLE we selected gene expression data for all cell lines linked to the 18 solid cancer types researched here, leaving 583 cell lines. We determined the median expression of each gene over all selected cell lines, which we considered as the gene expression of the pan-cancer cell type. We filtered for ligands and receptors with expression ≥ 10 TPM in at least one of the 25 cell types considered. Furthermore, we excluded ligands and receptors that were expressed by a cell type but not paired to another ligand or receptor in one of the other 24 considered cell types, resulting in 867 LR pairs. The 10 TPM threshold used in the Ramilowski paper for the CAGE data was based on known expression data from B cells. To confirm the suitability of the 10 TPM threshold for the CCLE RNA-seq data, we considered six healthy B cell datasets from two studies[113,114] by comparing the sets of LR pairs expressed in the Ramilowski CAGE data versus RNA-seq B cells considering different thresholds. The 10 TPM cutoff allowed the retrieval of ~80% B-cell-specific LRs expressed in the Ramilowski data, while resulting in the RNA-seq-specific expression of only 3% of the full LR set (data not shown).

Next, we assigned a patient-specific weight to the LR pairs. The LR pair weight was defined as the minimum of the $log_2(TPM+1)$ expression of the ligand and the receptor, theorizing that a pair has a weaker bond if one of the genes is expressed at a lower level. Certain LR pair features were assigned equal weights because of involving the same gene as either ligand or receptor in their interaction; thus these LR pairs were grouped, reducing the total number of features to 813 LR pairs.

Cell-cell interactions

The 24 TME cell types were combined in 12 aggregated cell types (Table S6). To assign a weight to the CC interactions, we considered the number of active LR pairs between each two pairs of the 13 considered cell types (12 TME-aggregated cell types and the additional pan-cancer cell type), for a total of 169 CC pairs. For each CC pair (sender → receiver cell), we considered an LR pair active only if the ligand was expressed in the sender cell and the receptor was expressed in the receiver cell, using the 10 TPM threshold as described above. For each LR pair we computed the frequency across the whole TCGA database, with the idea that more rare interactions are more relevant to discriminate patients. The CC score for each patient was then computed as the sum of the inverse of the frequency of all the active LR pairs.

Proteomics data

We used protein data for 18 solid tumors for a total of 5,394 patients. Data were downloaded via The Cancer Proteome Atlas Portal (https://tcpaportal.org). We used RPPA data labeled "Level 4 Pan-Can 32," including 200 proteins and 58 phosphoproteins for a total of 258 features. For RMTRL, protein features with any missing value in a specific cancer type were not considered (in the range from 24 to 47 proteins depending on the cancer type). This was not done for BEMKL, which can handle missing values.

Transcriptomics-based scores of immune response

The 14 published transcriptomics signatures of the immune response are summarized in Table S1. Among them, immune cytolytic activity[31] represents the level of two cytolytic effectors, granzyme A and perforin, which are overexpressed upon CD8+ T cell activation. Ock immune signature[115] is based on the expression of 105 genes associated with the response to immunotherapy with the MAGE-A3 antigen. Immunophenoscore[116] is calculated according to genes related to MHC molecules, immunomodulators, and effector and suppressor cells. IMPRES[92] is obtained through a logical comparison between the expressions of immune-checkpoint gene pairs. Roh immune score (Roh_IS)[117] is defined by a set of genes involved in immune activation in relation to tumor rejection. Chemokine signature (chemokines)[33] is based on a gene set associated with inflammation and immunity, which is able to predict host immune reaction and the formation of tumor-localized lymphoid structure. Davoli immune signature (Davoli_IS)[118] is derived from the expression of cytotoxic CD8+ T cell and NK cell markers. IFN-γ signature[34] comprises genes able to separate responders and non-responders in melanoma. Immune expanded signature (Ayes_expIS)[34] is generated by searching for genes highly correlated with IFN-γ signature genes; this new set included all immune-related genes. T cell inflamed microenvironment signature (T cell_inflamed)[34] is based on the joint potential of IFN-γ and T-cell-associated inflammatory genes in predicting response to PD-1 blockade. TIDE[119] is developed on the basis of immune escape signatures, such as T cell dysfunction or exclusion. MSI status[120] is determined by logical comparison of MSI-related gene pairs. TLS[32] signature is derived from differentially expressed genes in tumors with TLS. Repressed immune resistance[121] is defined by combining a set of gene signatures associated with T cell exclusion, post-treatment, and functional resistance.

All these proxies were calculated following the methodology reported by the original studies. For the chemokine signature only, we adjusted the sign according to the positive correlation with the cluster of correlated tasks. Since it is computed based on the first principal component, the sign is arbitrarily determined. When applicable, these transcriptional predictors were computed according to published computational frameworks. More details can be found in Table S1.

Machine learning methods

Regularized multi-task linear regression

The objective function that defines the RMTLR for N observations is described in Equation 1:

$$\frac{1}{N}\sum_{i=1}^{N}\|y_i-\beta_0-x_i\beta\|_2^2+\lambda\sum_{j=1}^{p}\left((1-\alpha)\|\beta_j\|_2+\alpha\|\beta_j\|_2^2\right). \quad \text{(Equation 1)}$$

In this equation, y_i represents a q-dimensional row vector where each entry corresponds to a task, and x_i is a row vector where each entry represents an observed feature. The aim of the RMTLR is to estimate a matrix β, whose rows represent the relation between one feature and all the tasks, and a vector β_0 of offsets (one for each task). The regularization term of RMTLR is a grouped version of the elastic net that aims at enforcing sparsity to entire rows of β.[28,122] In this way, the features corresponding to those rows of β that are set to zero do not contribute to the model. The strength of the regularization effect is tuned via the hyperparameter λ, while α regulates the interplay between the ridge- and the lasso-like terms of the elastic net. We selected the hyperparameters using 5-fold cross-validation.

We used RMTLR as implemented in the glmnet R package 2.0-16.[123] When applying RMTLR to a combination of multiple views, individual derived signatures (single views) were combined by merging datasets by columns.

Bayesian efficient multiple-kernel learning

BEMKL[29] is a Bayesian approach with two important features: multi-view and multi-task learning. BEMKL is a non-linear regression model that defines view-specific kernels as similarity measures between all samples and integrates them into a combined kernel to obtain response predictions (Equation 2). The similarity between samples is calculated using the Gaussian kernel.

On one hand, BEMKL uses multi-view learning to integrate different sample views as kernels, creating a combined kernel as the weighted sum of the view-specific kernels. The kernel weights were learned using multiple-kernel learning and represent the view's importance for predicting the response. On the other hand, the peculiarity of multi-task learning is that it enables one to model multiple tasks simultaneously. Assuming that the kernel weights are shared across all tasks, task-specific weights are estimated for all samples:

$$f(x) = a^T\left(\sum_{m=1}^{M} e_m k_m\left(x_i^m, x^m\right)\right) + b, \qquad \text{(Equation 2)}$$

where M denotes the number of input kernels and $k_m(x_i^m, x^m)$ represents the view-specific kernel, $e_m$the shared kernel weights, a the task-specific sample weights, and b the error term.

Bayesian inference was used to estimate all model parameters that were interpreted as random variables with certain probability distributions. The parameters of these distributions were learned using deterministic variational approximation. A more detailed explanation of the probabilistic model used and the inference method can be found in the original paper.[29,30] An R implementation of this method is available at https://github.com/mehmetgonen/bemkl. An adaptation to multi-task learning is available at https://github.com/mehr-een/bemkl-rbps, and we converted the code from MATLAB to R: https://github.com/olapuentesantana/mechanistic_biomarkers_immuno-oncology.

Model training based on TCGA data

Models were trained using TCGA data separately for each cancer type. For both RMTLR and BEMKL, training was repeated 100 times with randomized cross-validation, each time randomly picking 20% of the samples as a test set. This helped to assess the stability of the model, in terms of both performance and feature selection. For each iteration, we first standardized the training set, and then we standardized the test set based on the mean and standard deviation of the training set.

Definition of biomarkers of immune response

As mentioned above, the model training was repeated 100 times, resulting in an estimated 100 weight values for each biomarker. This allowed us to assess the stability of the features and assess whether the biomarkers were significantly different from zero. The values displayed in Figures 2, 3, and 4 were defined as the median of the estimated weights, first across runs and second across tasks (median >0, i.e., selected by regularization in at least 50% of the runs). Only statistically significant biomarkers (Wilcoxon rank-sum test, $p < 0.05$) are reported.

Prediction of response to ICB therapy using EaSIeR

All 100 models learned in the randomized cross-validation were included in the EaSIeR tool and were used to make predictions for the external test tests. For each validation dataset we used the corresponding cancer-type-specific model: SKCM for the melanoma Gide, Auslander, Riaz, and Liu cohorts; STAD for the gastric cancer Kim cohort; BLCA for the bladder cancer Mariathasan cohort; and GBM for the glioblastoma Cloughesy cohort. Predictions for each task were computed as the average of the 100 cancer-type-specific models. Prediction performances of cancer-type-specific models for each dataset were also compared with models built for the remaining 17 cancer types (Figure S5).

Integration of information on tumor mutational burden

For the Kim cohort we used the classification of low TMB (TMB_L), moderate TMB (TMB_M), and high TMB (TMB_H) as provided in the main text of the original publication.[96] For the other cohorts, patients were grouped in thirds as described in Carbone et al.[124] The lower tertile was considered as TMB_L, the intermediate as TMB_M, and the upper as TMB_H. Predictions of immune response using EaSIeR (using the ensemble model averaged across tasks) and information on tumor antigenicity based on the measured TMB were integrated using two different approaches.

In the first approach ("penalized score") the final prediction for each patient (P_i) is obtained by subtracting or adding a penalty (γ) to the score obtained using EaSIeR ($P_{EaSIeR,i}$), for patients with TMB_L or TMB_H, respectively, as described in Equation 3:

$$P_i = P_{EaSIeR,i} + c \cdot \gamma, \qquad \text{(Equation 3)}$$

where $c = -1$ if $TMB_i = TMB_L$, $c = 1$ if $TMB_i = TMB_H$, and $c = 0$ if $TMB_i = TMB_M$.

In the second approach ("weighted average") the final prediction for each patient (P_i) is obtained computing the weighted average between the score obtained using EaSIeR ($P_{EaSIeR,i}$) and the TMB. Both EaSIeR predictions and TMB were scaled between 0 and 1 to make them comparable. In this way $TMB_L = 0$, $TMB_M = 0.5$, and $TMB_H = 1$. The relative weight is given by the hyperparameter η as described in Equation 4:

$$P_i = (1 - \eta) \cdot P_{EaSIeR,i} + \eta \cdot TMB_i. \qquad \text{(Equation 4)}$$

Statistical analysis

We used Wilcoxon rank-sum test to assess whether the coefficients estimated for the biomarkers using the 100 randomized cross-validations were significantly different from zero. We used one-sided Wilcoxon signed-rank test (pair data) for comparison of predictions between pairwise combinations of derived signatures and single ones and for comparison of cross-cancer-type predictions. Two-sided Wilcoxon rank-sum test (unpaired data) was used for biomarker comparison between responders and non-responders. Statistical tests were carried out using the function "wilcox_test" from the R package rstatix version 0.6.0. Effect size was calculated as the test statistic divided by the square root of the number of observations, using the function 'wilcox_effsize' from the R package rstatix version 0.6.0.

Model performances were evaluated using the Spearman correlation for randomized cross-validation. For the validation dataset we computed the receiver operating characteristic curve and the AUC using the R package ROCR 1.0-11.

All analyses were performed using R software, version 4.0.2. For training of machine learning models, we used R software version 3.5.2.

SUPPLEMENTAL INFORMATION

Supplemental information can be found online at https://doi.org/10.1016/j.patter.2021.100293.

ACKNOWLEDGMENTS

We thank Federico Marini for useful discussion and feedback. F.F. was supported by the Austrian Science Fund (FWF) (project T 974-B30).

AUTHOR CONTRIBUTIONS

Conceptualization, F.E. and F.F.; methodology and formal analysis, O.L.S., M.G., F.F., and F.E.; software, O.L.S.; results interpretation, O.L.S., F.E., F.F., and P.A.J.H.; writing – original draft, F.E., O.L.S., and F.F.; writing – review & editing, O.L.S., M.G., P.A.J.H., F.F., and F.E.

CellPress
OPEN ACCESS

DECLARATION OF INTERESTS

The authors declare no competing interests.

Received: February 25, 2021
Revised: March 22, 2021
Accepted: May 31, 2021
Published: June 30, 2021

REFERENCES

1. Robert, C. (2020). A decade of immune-checkpoint inhibitors in cancer therapy. Nat. Commun. *11*, 3801.
2. Boutros, C., Tarhini, A., Routier, E., Lambotte, O., Ladurie, F.L., Carbonnel, F., Izzeddine, H., Marabelle, A., Champiat, S., Berdelou, A., et al. (2016). Safety profiles of anti-CTLA-4 and anti-PD-1 antibodies alone and in combination. Nat. Rev. Clin. Oncol. *13*, 473–486.
3. Postow, M.A., Sidlow, R., and Hellmann, M.D. (2018). Immune-related adverse events associated with immune checkpoint blockade. N. Engl. J. Med. *378*, 158–168.
4. Schmidt, C. (2017). The benefits of immunotherapy combinations. Nature *552*, S67–S69.
5. Tang, J., Pearce, L., O'Donnell-Tormey, J., and Hubbard-Lucey, V.M. (2018). Trends in the global immuno-oncology landscape. Nat. Rev. Drug Discov. *17*, 922.
6. Lapuente-Santana, Ó., and Eduati, F. (2020). Toward systems biomarkers of response to immune checkpoint blockers. Front. Oncol. *10*, 1027.
7. Fridman, W.H., Zitvogel, L., Sautès-Fridman, C., and Kroemer, G. (2017). The immune contexture in cancer prognosis and treatment. Nat. Rev. Clin. Oncol. *14*, 717–734.
8. Galon, J., and Bruni, D. (2019). Approaches to treat immune hot, altered and cold tumours with combination immunotherapies. Nat. Rev. Drug Discov. *18*, 197–218.
9. Spranger, S., and Gajewski, T.F. (2018). Impact of oncogenic pathways on evasion of antitumour immune responses. Nat. Rev. Cancer *18*, 139–147.
10. Zerdes, I., Matikas, A., Bergh, J., Rassidakis, G.Z., and Foukakis, T. (2018). Genetic, transcriptional and post-translational regulation of the programmed death protein ligand 1 in cancer: biology and clinical correlations. Oncogene *37*, 4639–4661.
11. Tokunaga, R., Zhang, W., Naseem, M., Puccini, A., Berger, M.D., Soni, S., McSkane, M., Baba, H., and Lenz, H.-J. (2018). CXCL9, CXCL10, CXCL11/CXCR3 axis for immune activation - a target for novel cancer therapy. Cancer Treat. Rev. *63*, 40–47.
12. Nagarsheth, N., Wicha, M.S., and Zou, W. (2017). Chemokines in the cancer microenvironment and their relevance in cancer immunotherapy. Nat. Rev. Immunol. *17*, 559–572.
13. Cornel, A.M., Mimpen, I.L., and Nierkens, S. (2020). MHC class I downregulation in cancer: underlying mechanisms and potential targets for cancer immunotherapy. Cancers *12*, 1760.
14. Ramilowski, J.A., Goldberg, T., Harshbarger, J., Kloppmann, E., Lizio, M., Satagopam, V.P., Itoh, M., Kawaji, H., Carninci, P., Rost, B., et al. (2015). A draft network of ligand-receptor-mediated multicellular signalling in human. Nat. Commun. *6*, 7866.
15. Kumar, M.P., Du, J., Lagoudas, G., Jiao, Y., Sawyer, A., Drummond, D.C., Lauffenburger, D.A., and Raue, A. (2018). Analysis of single-cell RNA-seq identifies cell-cell communication associated with tumor characteristics. Cell Rep. *25*, 1458–1468.e4.
16. Szeto, G.L., and Finley, S.D. (2019). Integrative approaches to cancer immunotherapy. Trends Cancer Res. *5*, 400–410.
17. Finotello, F., and Eduati, F. (2018). Multi-omics profiling of the tumor microenvironment: paving the way to precision immuno-oncology. Front. Oncol. *8*, 430.
18. Finotello, F., Rieder, D., Hackl, H., and Trajanoski, Z. (2019). Next-generation computational tools for interrogating cancer immunity. Nat. Rev. Genet. *20*, 724–746.
19. Stark, R., Grzelak, M., and Hadfield, J. (2019). RNA sequencing: the teenage years. Nat. Rev. Genet. *20*, 631–656.
20. Weinstein, J.N., Collisson, E.A., Mills, G.B., Mills Shaw, K.R., Ozenberger, B.A., Ellrott, K., Shmulevich, I., Sander, C., and Stuart, J.M. (2013). The cancer Genome Atlas pan-cancer analysis project. Nat. Genet. *45*, 1113–1120.
21. Byron, S.A., Van Keuren-Jensen, K.R., Engelthaler, D.M., Carpten, J.D., and Craig, D.W. (2016). Translating RNA sequencing into clinical diagnostics: opportunities and challenges. Nat. Rev. Genet. *17*, 257–271.
22. Finotello, F., and Trajanoski, Z. (2018). Quantifying tumor-infiltrating immune cells from transcriptomics data. Cancer Immunol. Immunother. *67*, 1031–1040.
23. Schubert, M., Klinger, B., Klünemann, M., Sieber, A., Uhlitz, F., Sauer, S., Garnett, M.J., Blüthgen, N., and Saez-Rodriguez, J. (2018). Perturbation-response genes reveal signaling footprints in cancer gene expression. Nat. Commun. *9*, 20.
24. Garcia-Alonso, L., Iorio, F., Matchan, A., Fonseca, N., Jaaks, P., Peat, G., Pignatelli, M., Falcone, F., Benes, C.H., Dunham, I., et al. (2018). Transcription factor Activities enhance markers of drug sensitivity in cancer. Cancer Res. *78*, 769–780.
25. Finotello, F., Mayer, C., Plattner, C., Laschober, G., Rieder, D., Hackl, H., Krogsdam, A., Loncova, Z., Posch, W., Wilflingseder, D., et al. (2019). Molecular and pharmacological modulators of the tumor immune contexture revealed by deconvolution of RNA-seq data. Genome Med. *11*, 34.
26. Holland, C.H., Szalai, B., and Saez-Rodriguez, J. (2020). Transfer of regulatory knowledge from human to mouse for functional genomics analysis. Biochim. Biophys. Acta Gene Regul. Mech. *1863*, 194431.
27. Efremova, M., Vento-Tormo, M., Teichmann, S.A., and Vento-Tormo, R. (2020). CellPhoneDB: inferring cell-cell communication from combined expression of multi-subunit ligand-receptor complexes. Nat. Protoc. *15*, 1484–1506.
28. Yuan, M., and Lin, Y. (2006). Model selection and estimation in regression with grouped variables. J. R. Stat. Soc. B Stat. Methodol. *68*, 49–67.
29. Gönen, M. (2012). Bayesian efficient multiple kernel learning. In Proceedings of the 29th International Conference on Machine Learning (ICML 2012)), pp. 1–8.
30. Costello, J.C., Heiser, L.M., Georgii, E., Gönen, M., Menden, M.P., Wang, N.J., Bansal, M., Ammad-ud-din, M., Hintsanen, P., Khan, S.A., et al. (2014). A community effort to assess and improve drug sensitivity prediction algorithms. Nat. Biotechnol. *32*, 1202.
31. Rooney, M.S., Shukla, S.A., Wu, C.J., Getz, G., and Hacohen, N. (2015). Molecular and genetic properties of tumors associated with local immune cytolytic activity. Cell *160*, 48–61.
32. Cabrita, R., Lauss, M., Sanna, A., Donia, M., Skaarup Larsen, M., Mitra, S., Johansson, I., Phung, B., Harbst, K., Vallon-Christersson, J., et al. (2020). Tertiary lymphoid structures improve immunotherapy and survival in melanoma. Nature *577*, 561–565.
33. Messina, J.L., Fenstermacher, D.A., Eschrich, S., Qu, X., Berglund, A.E., Lloyd, M.C., Schell, M.J., Sondak, V.K., Weber, J.S., and Mulé, J.J. (2012). 12-Chemokine gene signature identifies lymph node-like structures in melanoma: potential for patient selection for immunotherapy? Sci. Rep. *2*, 765.
34. Ayers, M., Lunceford, J., Nebozhyn, M., Murphy, E., Loboda, A., Kaufman, D.R., Albright, A., Cheng, J.D., Kang, S.P., Shankaran, V., et al. (2017). IFN-γ-related mRNA profile predicts clinical response to PD-1 blockade. J. Clin. Invest. *127*, 2930–2940.
35. Chen, D.S., and Mellman, I. (2013). Oncology meets immunology: the cancer-immunity cycle. Immunity *39*, 1–10.
36. Helmink, B.A., Reddy, S.M., Gao, J., Zhang, S., Basar, R., Thakur, R., Yizhak, K., Sade-Feldman, M., Blando, J., Han, G., et al. (2020). B cells

and tertiary lymphoid structures promote immunotherapy response. Nature *577*, 549–555.

37. Johnstone, R.W., Frew, A.J., and Smyth, M.J. (2008). The TRAIL apoptotic pathway in cancer onset, progression and therapy. Nat. Rev. Cancer *8*, 782–798.
38. Jorgovanovic, D., Song, M., Wang, L., and Zhang, Y. (2020). Roles of IFN-γ in tumor progression and regression: a review. Biomark Res. *8*, 49.
39. Ding, Q., Lu, P., Xia, Y., Ding, S., Fan, Y., Li, X., Han, P., Liu, J., Tian, D., and Liu, M. (2016). CXCL9: evidence and contradictions for its role in tumor progression. Cancer Med. *5*, 3246–3259.
40. Parsa, A.T., Waldron, J.S., Panner, A., Crane, C.A., Parney, I.F., Barry, J.J., Cachola, K.E., Murray, J.C., Tihan, T., Jensen, M.C., et al. (2007). Loss of tumor suppressor PTEN function increases B7-H1 expression and immunoresistance in glioma. Nat. Med. *13*, 84–88.
41. Vivanco, I., and Sawyers, C.L. (2002). The phosphatidylinositol 3-Kinase–AKT pathway in human cancer. Nat. Rev. Cancer *2*, 489–501.
42. Chen, D.S., and Mellman, I. (2017). Elements of cancer immunity and the cancer-immune set point. Nature *541*, 321–330.
43. Bonaventura, P., Shekarian, T., Alcazer, V., Valladeau-Guilemond, J., Valsesia-Wittmann, S., Amigorena, S., Caux, C., and Depil, S. (2019). Cold tumors: a therapeutic challenge for immunotherapy. Front. Immunol. *10*, 168.
44. Manegold, C., Dingemans, A.-M.C., Gray, J.E., Nakagawa, K., Nicolson, M., Peters, S., Reck, M., Wu, Y.-L., Brustugun, O.T., Crinò, L., et al. (2017). The potential of combined immunotherapy and antiangiogenesis for the synergistic treatment of advanced NSCLC. J. Thorac. Oncol. *12*, 194–207.
45. Dong, Z.-Y., Zhong, W.-Z., Zhang, X.-C., Su, J., Xie, Z., Liu, S.-Y., Tu, H.-Y., Chen, H.-J., Sun, Y.-L., Zhou, Q., et al. (2017). Potential predictive value of TP53 and KRAS mutation status for response to PD-1 blockade immunotherapy in lung adenocarcinoma. Clin. Cancer Res. *23*, 3012–3024.
46. Qin, Z., Zhang, X., Chen, Z., and Liu, N. (2020). Establishment and validation of an immune-based prognostic score model in glioblastoma. Int. Immunopharmacol. *85*, 106636.
47. Noman, M.Z., Hasmim, M., Lequeux, A., Xiao, M., Duhem, C., Chouaib, S., Berchem, G., and Janji, B. (2019). Improving cancer immunotherapy by targeting the hypoxic tumor microenvironment: new opportunities and challenges. Cells *8*, 1083.
48. Jayaprakash, P., Ai, M., Liu, A., Budhani, P., Bartkowiak, T., Sheng, J., Ager, C., Nicholas, C., Jaiswal, A.R., Sun, Y., et al. (2018). Targeted hypoxia reduction restores T cell infiltration and sensitizes prostate cancer to immunotherapy. J. Clin. Invest. *128*, 5137–5149.
49. Yu, S., Liu, D., Shen, B., Shi, M., and Feng, J. (2018). Immunotherapy strategy of EGFR mutant lung cancer. Am. J. Cancer Res. *8*, 2106–2115.
50. Garcia-Diaz, A., Shin, D.S., Moreno, B.H., Saco, J., Escuin-Ordinas, H., Rodriguez, G.A., Zaretsky, J.M., Sun, L., Hugo, W., Wang, X., et al. (2019). Interferon receptor signaling pathways regulating PD-L1 and PD-L2 expression. Cell Rep. *29*, 3766.
51. Zou, S., Tong, Q., Liu, B., Huang, W., Tian, Y., and Fu, X. (2020). Targeting STAT3 in cancer immunotherapy. Mol. Cancer *19*, 145.
52. Bellucci, R., Martin, A., Bommarito, D., Wang, K., Hansen, S.H., Freeman, G.J., and Ritz, J. (2015). Interferon-γ-induced activation of JAK1 and JAK2 suppresses tumor cell susceptibility to NK cells through upregulation of PD-L1 expression. Oncoimmunology *4*, e1008824.
53. Chen, S., Crabill, G.A., Pritchard, T.S., McMiller, T.L., Wei, P., Pardoll, D.M., Pan, F., and Topalian, S.L. (2019). Mechanisms regulating PD-L1 expression on tumor and immune cells. J. Immunother. Cancer *7*, 305.
54. Anderson, K., Ryan, N., Volpedo, G., Varikuti, S., Satoskar, A.R., and Oghumu, S. (2019). Immune suppression mediated by STAT4 deficiency promotes lymphatic metastasis in HNSCC. Front. Immunol. *10*, 3095.
55. Nishi, M., Batsaikhan, B.-E., Yoshikawa, K., Higashijima, J., Tokunaga, T., Takasu, C., Kashihara, H., Ishikawa, D., and Shimada, M. (2017). High STAT4 expression indicates better disease-free survival in patients with gastric cancer. Anticancer Res. *37*, 6723–6729.
56. Lee, S.-J., Jang, B.-C., Lee, S.-W., Yang, Y.-I., Suh, S.-I., Park, Y.-M., Oh, S., Shin, J.-G., Yao, S., Chen, L., et al. (2006). Interferon regulatory factor-1 is prerequisite to the constitutive expression and IFN-gamma-induced upregulation of B7-H1 (CD274). FEBS Lett. *580*, 755–762.
57. Gowrishankar, K., Gunatilake, D., Gallagher, S.J., Tiffen, J., Rizos, H., and Hersey, P. (2015). Inducible but not constitutive expression of PD-L1 in human melanoma cells is dependent on activation of NF-κB. PLoS One *10*, e0123410.
58. Galluzzi, L., Chan, T.A., Kroemer, G., Wolchok, J.D., and López-Soto, A. (2018). The hallmarks of successful anticancer immunotherapy. Sci. Transl. Med. *10*, eaat7807.
59. Meissner, T.B., Liu, Y.-J., Lee, K.-H., Li, A., Biswas, A., van Eggermond, M.C.J.A., van den Elsen, P.J., and Kobayashi, K.S. (2012). NLRC5 cooperates with the RFX transcription factor complex to induce MHC class I gene expression. J. Immunol. *188*, 4951–4958.
60. Yoshihama, S., Vijayan, S., Sidiq, T., and Kobayashi, K.S. (2017). NLRC5/CITA: a key player in cancer immune surveillance. Trends Cancer Res. *3*, 28–38.
61. Chelbi, S.T., and Guarda, G. (2016). NLRC5, a promising new entry in tumor immunology. J. Immunother. Cancer *4*, 39.
62. Manandhar, S., and Lee, Y.M. (2018). Emerging role of RUNX3 in the regulation of tumor microenvironment. BMB Rep. *51*, 174–181.
63. Aasebø, K., Dragomir, A., Sundström, M., Mezheyeuski, A., Edqvist, P.-H., Eide, G.E., Ponten, F., Pfeiffer, P., Glimelius, B., and Sorbye, H. (2020). CDX2: a prognostic marker in metastatic colorectal cancer defining a better BRAF mutated and a worse KRAS mutated subgroup. Front. Oncol. *10*, 8.
64. Inaguma, S., Lasota, J., Wang, Z., Felisiak-Golabek, A., Ikeda, H., and Miettinen, M. (2017). Clinicopathologic profile, immunophenotype, and genotype of CD274 (PD-L1)-positive colorectal carcinomas. Mod. Pathol. *30*, 278–285.
65. Bougarne, N., Weyers, B., Desmet, S.J., Deckers, J., Ray, D.W., Staels, B., and De Bosscher, K. (2018). Molecular actions of PPARα in lipid metabolism and inflammation. Endocr. Rev. *39*, 760–802.
66. Laport, G., Powderly, J.D., Chokshi, S., Luke, J.J., Bendell, J.C., Enstrom, A., Whiting, C.C., and Dubensky, T.W. (2019). Phase 1/1b multicenter trial of TPST-1120, a peroxisome proliferator-activated receptor alpha (PPARα) antagonist as a single agent (SA) or in combination in patients with advanced solid tumors. J. Clin. Oncol. *37*, TPS2665.
67. Li, J., Lu, Y., Akbani, R., Ju, Z., Roebuck, P.L., Liu, W., Yang, J.-Y., Broom, B.M., Verhaak, R.G.W., Kane, D.W., et al. (2013). TCPA: a resource for cancer functional proteomics data. Nat. Methods *10*, 1046–1047.
68. Jiang, D., Liang, J., Campanella, G.S., Guo, R., Yu, S., Xie, T., Liu, N., Jung, Y., Homer, R., Meltzer, E.B., et al. (2010). Inhibition of pulmonary fibrosis in mice by CXCL10 requires glycosaminoglycan binding and syndecan-4. J. Clin. Invest. *120*, 2049–2057.
69. Barrett, R., and Puré, E. (2020). Cancer-associated fibroblasts: key determinants of tumor immunity and immunotherapy. Curr. Opin. Immunol. *64*, 80–87.
70. Aldinucci, D., Borghese, C., and Casagrande, N. (2020). The CCL5/CCR5 axis in cancer progression. Cancers *12*, 1765.
71. Dangaj, D., Bruand, M., Grimm, A.J., Ronet, C., Barras, D., Duttagupta, P.A., Lanitis, E., Duraiswamy, J., Tanyi, J.L., Benencia, F., et al. (2019). Cooperation between constitutive and inducible chemokines enables T cell engraftment and immune attack in solid tumors. Cancer Cell *35*, 885–900.e10.
72. Kim, M.J., Sun, H.J., Song, Y.S., Yoo, S.-K., Kim, Y.A., Seo, J.-S., Park, Y.J., and Cho, S.W. (2019). CXCL16 positively correlated with M2-macrophage infiltration, enhanced angiogenesis, and poor prognosis in thyroid cancer. Sci. Rep. *9*, 13288.

CellPress
OPEN ACCESS

73. Hojo, S., Koizumi, K., Tsuneyama, K., Arita, Y., Cui, Z., Shinohara, K., Minami, T., Hashimoto, I., Nakayama, T., Sakurai, H., et al. (2007). High-level expression of chemokine CXCL16 by tumor cells correlates with a good prognosis and increased tumor-infiltrating lymphocytes in colorectal cancer. Cancer Res. *67*, 4725–4731.

74. Yoon, M.S., Pham, C.T., Phan, M.-T.T., Shin, D.-J., Jang, Y.-Y., Park, M.-H., Kim, S.-K., Kim, S., and Cho, D. (2016). Irradiation of breast cancer cells enhances CXCL16 ligand expression and induces the migration of natural killer cells expressing the CXCR6 receptor. Cytotherapy *18*, 1532–1542.

75. Sharma, P., Hu-Lieskovan, S., Wargo, J.A., and Ribas, A. (2017). Primary, adaptive, and acquired resistance to cancer immunotherapy. Cell *168*, 707–723.

76. Gao, J., Shi, L.Z., Zhao, H., Chen, J., Xiong, L., He, Q., Chen, T., Roszik, J., Bernatchez, C., Woodman, S.E., et al. (2016). Loss of IFN-γ pathway genes in tumor cells as a mechanism of resistance to anti-CTLA-4 therapy. Cell *167*, 397–404.e9.

77. Motyka, B., Korbutt, G., Pinkoski, M.J., Heibein, J.A., Caputo, A., Hobman, M., Barry, M., Shostak, I., Sawchuk, T., Holmes, C.F.B., et al. (2000). Mannose 6-Phosphate/Insulin-like growth factor II receptor is a death receptor for granzyme B during cytotoxic T cell–induced apoptosis. Cell *103*, 491–500.

78. Li, C., Zeng, M., Chi, H., Shen, J., Ng, T.-B., Jin, G., Lu, D., Fan, X., Xiong, B., Xiao, Z., et al. (2017). Trichosanthin increases Granzyme B penetration into tumor cells by upregulation of CI-MPR on the cell surface. Oncotarget *8*, 26460–26470.

79. Modiano, J.F., and Bellgrau, D. (2016). Fas ligand based immunotherapy: a potent and effective neoadjuvant with checkpoint inhibitor properties, or a systemically toxic promoter of tumor growth? Discov. Med. *21*, 109–116.

80. Serriari, N.-E., Gondois-Rey, F., Guillaume, Y., Remmerswaal, E.B.M., Pastor, S., Messal, N., Truneh, A., Hirsch, I., van Lier, R.A.W., and Olive, D. (2010). B and T lymphocyte attenuator is highly expressed on CMV-specific T cells during infection and regulates their function. J. Immunol. *185*, 3140–3148.

81. Haymaker, C.L., Wu, R.C., Ritthipichai, K., Bernatchez, C., Forget, M.-A., Chen, J.Q., Liu, H., Wang, E., Marincola, F., Hwu, P., et al. (2015). BTLA marks a less-differentiated tumor-infiltrating lymphocyte subset in melanoma with enhanced survival properties. Oncoimmunology *4*, e1014246.

82. van de Ven, K., and Borst, J. (2015). Targeting the T-cell co-stimulatory CD27/CD70 pathway in cancer immunotherapy: rationale and potential. Immunotherapy *7*, 655–667.

83. André, P., Denis, C., Soulas, C., Bourbon-Caillet, C., Lopez, J., Arnoux, T., Bléry, M., Bonnafous, C., Gauthier, L., Morel, A., et al. (2018). Anti-NKG2A mAb is a checkpoint inhibitor that promotes anti-tumor immunity by unleashing both T and NK cells. Cell *175*, 1731–1743.e13.

84. Del Campo, A.B., Carretero, J., Muñoz, J.A., Zinchenko, S., Ruiz-Cabello, F., González-Aseguinolaza, G., Garrido, F., and Aptsiauri, N. (2014). Adenovirus expressing β2-microglobulin recovers HLA class I expression and antitumor immunity by increasing T-cell recognition. Cancer Gene Ther. *21*, 317–332.

85. Reina, M., and Espel, E. (2017). Role of LFA-1 and ICAM-1 in cancer. Cancers *9*, 153.

86. Yanguas, A., Garasa, S., Teijeira, Á., Aubá, C., Melero, I., and Rouzaut, A. (2018). ICAM-1-LFA-1 dependent CD8+ T-lymphocyte aggregation in tumor tissue prevents recirculation to draining lymph nodes. Front. Immunol. *9*, 2084.

87. Klein, D. (2018). The tumor vascular endothelium as decision maker in cancer therapy. Front. Oncol. *8*, 367.

88. Raskov, H., Orhan, A., Christensen, J.P., and Gögenur, I. (2021). Cytotoxic CD8 T cells in cancer and cancer immunotherapy. Br. J. Cancer *124*, 359–367.

89. Blanca, I.R., Bere, E.W., Young, H.A., and Ortaldo, J.R. (2001). Human B cell activation by autologous NK cells is regulated by CD40-CD40 ligand interaction: role of memory B cells and CD5+ B cells. J. Immunol. *167*, 6132–6139.

90. Parsa, R., Lund, H., Georgoudaki, A.-M., Zhang, X.-M., Ortlieb Guerreiro-Cacais, A., Grommisch, D., Warnecke, A., Croxford, A.L., Jagodic, M., Becher, B., et al. (2016). BAFF-secreting neutrophils drive plasma cell responses during emergency granulopoiesis. J. Exp. Med. *213*, 1537–1553.

91. Gide, T.N., Quek, C., Menzies, A.M., Tasker, A.T., Shang, P., Holst, J., Madore, J., Lim, S.Y., Velickovic, R., Wongchenko, M., et al. (2019). Distinct immune cell populations define response to anti-PD-1 monotherapy and anti-PD-1/anti-CTLA-4 combined therapy. Cancer Cell *35*, 238–255.e6.

92. Auslander, N., Zhang, G., Lee, J.S., Frederick, D.T., Miao, B., Moll, T., Tian, T., Wei, Z., Madan, S., Sullivan, R.J., et al. (2018). Robust prediction of response to immune checkpoint blockade therapy in metastatic melanoma. Nat. Med. *24*, 1545–1549.

93. Riaz, N., Havel, J.J., Makarov, V., Desrichard, A., Urba, W.J., Sims, J.S., Hodi, F.S., Martín-Algarra, S., Mandal, R., Sharfman, W.H., et al. (2017). Tumor and microenvironment evolution during immunotherapy with nivolumab. Cell *171*, 934–949.e15.

94. Liu, D., Schilling, B., Liu, D., Sucker, A., Livingstone, E., Jerby-Arnon, L., Zimmer, L., Gutzmer, R., Satzger, I., Loquai, C., et al. (2019). Integrative molecular and clinical modeling of clinical outcomes to PD1 blockade in patients with metastatic melanoma. Nat. Med. *25*, 1916–1927.

95. Mariathasan, S., Turley, S.J., Nickles, D., Castiglioni, A., Yuen, K., Wang, Y., Kadel, E.E., III, Koeppen, H., Astarita, J.L., Cubas, R., et al. (2018). TGFβ attenuates tumour response to PD-L1 blockade by contributing to exclusion of T cells. Nature *554*, 544–548.

96. Kim, S.T., Cristescu, R., Bass, A.J., Kim, K.-M., Odegaard, J.I., Kim, K., Liu, X.Q., Sher, X., Jung, H., Lee, M., et al. (2018). Comprehensive molecular characterization of clinical responses to PD-1 inhibition in metastatic gastric cancer. Nat. Med. *24*, 1449–1458.

97. Cloughesy, T.F., Mochizuki, A.Y., Orpilla, J.R., Hugo, W., Lee, A.H., Davidson, T.B., Wang, A.C., Ellingson, B.M., Rytlewski, J.A., Sanders, C.M., et al. (2019). Neoadjuvant anti-PD-1 immunotherapy promotes a survival benefit with intratumoral and systemic immune responses in recurrent glioblastoma. Nat. Med. *25*, 477–486.

98. Du, W., and Elemento, O. (2014). Cancer systems biology: embracing complexity to develop better anticancer therapeutic strategies. Oncogene *34*, 3215–3225.

99. Senft, D., Leiserson, M.D.M., Ruppin, E., and Ronai, Z.A. (2017). Precision oncology: the road ahead. Trends Mol. Med. *23*, 874–898.

100. Garcia-Alonso, L., Holland, C.H., Ibrahim, M.M., Turei, D., and Saez-Rodriguez, J. (2019). Benchmark and integration of resources for the estimation of human transcription factor activities. Genome Res. *29*, 1363–1375.

101. Sharma, P., and Allison, J.P. (2015). Immune checkpoint targeting in cancer therapy: toward combination strategies with curative potential. Cell *161*, 205–214.

102. Wu, Y., Chen, W., Xu, Z.P.G., and Gu, W. (2019). PD-L1 distribution and perspective for cancer immunotherapy–blockade, knockdown, or inhibition. Front. Immunol. *10*, 2022.

103. Coelho, M.A., de Carné Trécesson, S., Rana, S., Zecchin, D., Moore, C., Molina-Arcas, M., East, P., Spencer-Dene, B., Nye, E., Barnouin, K., et al. (2017). Oncogenic RAS signaling promotes tumor immunoresistance by stabilizing PD-L1 mRNA. Immunity *47*, 1083–1099.e6.

104. Armingol, E., Officer, A., Harismendy, O., and Lewis, N.E. (2021). Deciphering cell-cell interactions and communication from gene expression. Nat. Rev. Genet. *22*, 71–88.

105. Saltz, J., Gupta, R., Hou, L., Kurc, T., Singh, P., Nguyen, V., Samaras, D., Shroyer, K.R., Zhao, T., Batiste, R., et al. (2018). Spatial Organization and molecular correlation of tumor-infiltrating lymphocytes using deep learning on pathology images. Cell Rep. *23*, 181–193.e7.

106. Fu, Y., Jung, A.W., Torne, R.V., Gonzalez, S., and Vöhringer, H. (2020). Pan-cancer computational histopathology reveals mutations, tumor composition and prognosis. Nat. Cancer *1*, 800–810.

107. Jin, S., Guerrero-Juarez, C.F., Zhang, L., Chang, I., Ramos, R., Kuan, C.-H., Myung, P., Plikus, M.V., and Nie, Q. (2021). Inference and analysis of cell-cell communication using CellChat. Nat. Commun. *12*, 1088.

108. Holland, C.H., Tanevski, J., Perales-Patón, J., Gleixner, J., Kumar, M.P., Mereu, E., Joughin, B.A., Stegle, O., Lauffenburger, D.A., Heyn, H., et al. (2020). Robustness and applicability of transcription factor and pathway analysis tools on single-cell RNA-seq data. Genome Biol. *21*, 36.

109. Bolger, A.M., Lohse, M., and Usadel, B. (2014). Trimmomatic: a flexible trimmer for Illumina sequence data. Bioinformatics *30*, 2114–2120.

110. Bray, N.L., Pimentel, H., Melsted, P., and Pachter, L. (2016). Near-optimal probabilistic RNA-seq quantification. Nat. Biotechnol. *34*, 525–527.

111. Bonavita, E., Bromley, C.P., Jonsson, G., Pelly, V.S., Sahoo, S., Walwyn-Brown, K., Mensurado, S., Moeini, A., Flanagan, E., Bell, C.R., et al. (2020). Antagonistic inflammatory phenotypes dictate tumor fate and response to immune checkpoint blockade. Immunity *53*, 1215–1229.e8.

112. Barretina, J., Caponigro, G., Stransky, N., Venkatesan, K., Margolin, A.A., Kim, S., Wilson, C.J., Lehár, J., Kryukov, G.V., Sonkin, D., et al. (2012). The Cancer Cell Line Encyclopedia enables predictive modelling of anticancer drug sensitivity. Nature *483*, 603–607.

113. Linsley, P.S., Speake, C., Whalen, E., and Chaussabel, D. (2014). Copy number loss of the interferon gene cluster in melanomas is linked to reduced T cell infiltrate and poor patient prognosis. PLoS One *9*, e109760.

114. Hoek, K.L., Samir, P., Howard, L.M., Niu, X., Prasad, N., Galassie, A., Liu, Q., Allos, T.M., Floyd, K.A., Guo, Y., et al. (2015). A cell-based systems biology assessment of human blood to monitor immune responses after influenza vaccination. PLoS One *10*, e0118528.

115. Ock, C.-Y., Hwang, J.-E., Keam, B., Kim, S.-B., Shim, J.-J., Jang, H.-J., Park, S., Sohn, B.H., Cha, M., Ajani, J.A., et al. (2017). Genomic landscape associated with potential response to anti-CTLA-4 treatment in cancers. Nat. Commun. *8*, 1050.

116. Charoentong, P., Finotello, F., Angelova, M., Mayer, C., Efremova, M., Rieder, D., Hackl, H., and Trajanoski, Z. (2017). Pan-cancer Immunogenomic analyses reveal genotype-immunophenotype relationships and predictors of response to checkpoint blockade. Cell Rep. *18*, 248–262.

117. Roh, W., Chen, P.-L., Reuben, A., Spencer, C.N., Prieto, P.A., Miller, J.P., Gopalakrishnan, V., Wang, F., Cooper, Z.A., Reddy, S.M., et al. (2017). Integrated molecular analysis of tumor biopsies on sequential CTLA-4 and PD-1 blockade reveals markers of response and resistance. Sci. Transl. Med. *9*, eaah3560.

118. Davoli, T., Uno, H., Wooten, E.C., and Elledge, S.J. (2017). Tumor aneuploidy correlates with markers of immune evasion and with reduced response to immunotherapy. Science *355*, eaaf8399.

119. Jiang, P., Gu, S., Pan, D., Fu, J., Sahu, A., Hu, X., Li, Z., Traugh, N., Bu, X., Li, B., et al. (2018). Signatures of T cell dysfunction and exclusion predict cancer immunotherapy response. Nat. Med. *24*, 1550–1558.

120. Fu, Y., Qi, L., Guo, W., Jin, L., Song, K., You, T., Zhang, S., Gu, Y., Zhao, W., and Guo, Z. (2019). A qualitative transcriptional signature for predicting microsatellite instability status of right-sided Colon Cancer. BMC Genomics *20*, 769.

121. Jerby-Arnon, L., Shah, P., Cuoco, M.S., Rodman, C., Su, M.-J., Melms, J.C., Leeson, R., Kanodia, A., Mei, S., Lin, J.-R., et al. (2018). A cancer cell program promotes T cell exclusion and resistance to checkpoint blockade. Cell *175*, 984–997.e24.

122. Zou, H., and Hastie, T. (2005). Regularization and variable selection via the elastic net. J. R. Stat. Soc. B Stat. Methodol. *67*, 301–320.

123. Friedman, J., Hastie, T., and Tibshirani, R. (2010). Regularization paths for generalized linear models via coordinate descent. J. Stat. Softw. *33*, 1–22.

124. Carbone, D.P., Reck, M., Paz-Ares, L., Creelan, B., Horn, L., Steins, M., Felip, E., van den Heuvel, M.M., Ciuleanu, T.-E., Badin, F., et al. (2017). First-line nivolumab in stage IV or recurrent non-small-cell lung cancer. N. Engl. J. Med. *376*, 2415–2426.

Patterns, Volume *2*

Supplemental information

Interpretable systems biomarkers predict response to immune-checkpoint inhibitors

Óscar Lapuente-Santana, Maisa van Genderen, Peter A.J. Hilbers, Francesca Finotello, and Federica Eduati

SUPPLEMENTAL FIGURES

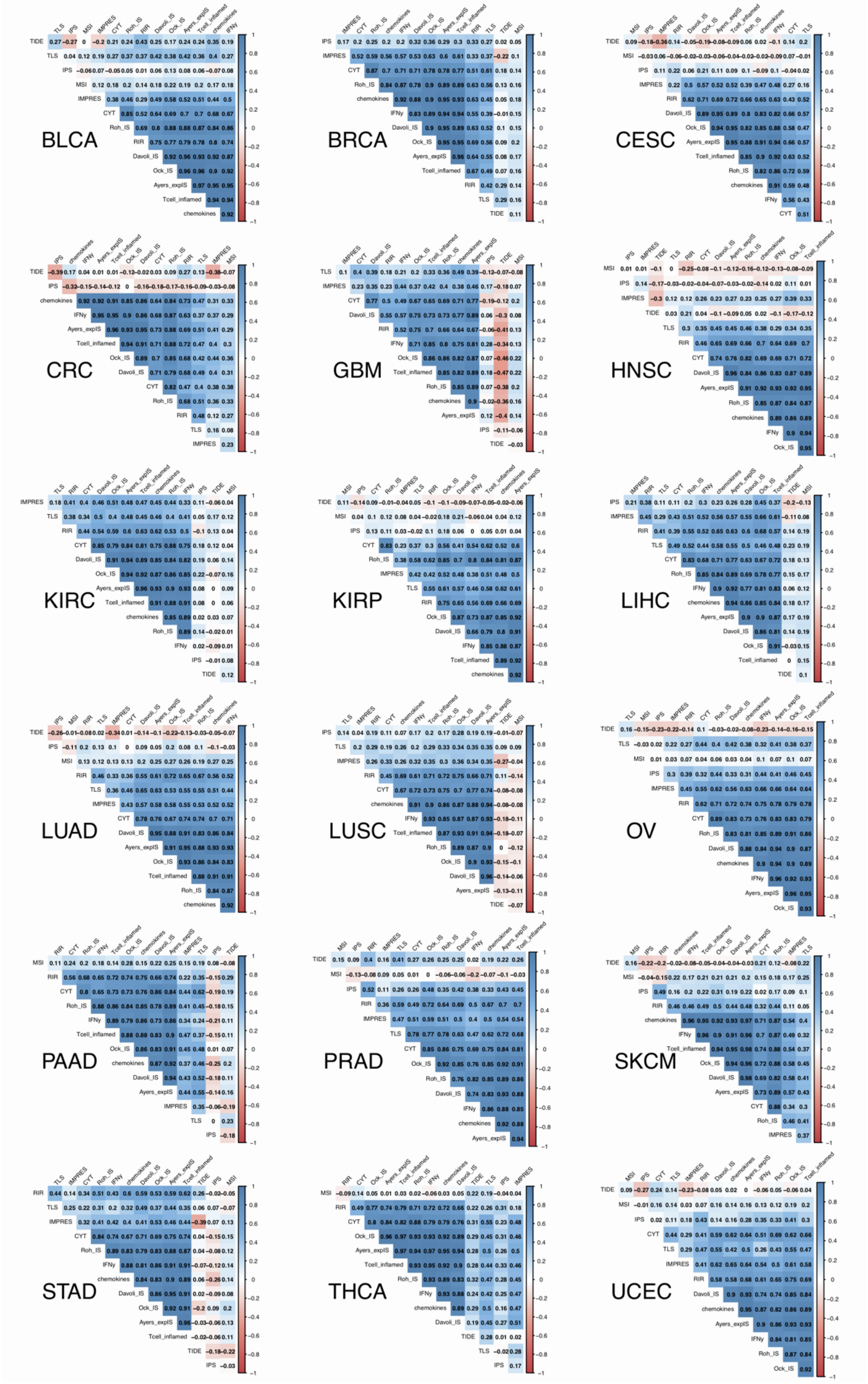

Figure S1. Cancer-specific Pearson correlations between the 14 proxies of immune response.

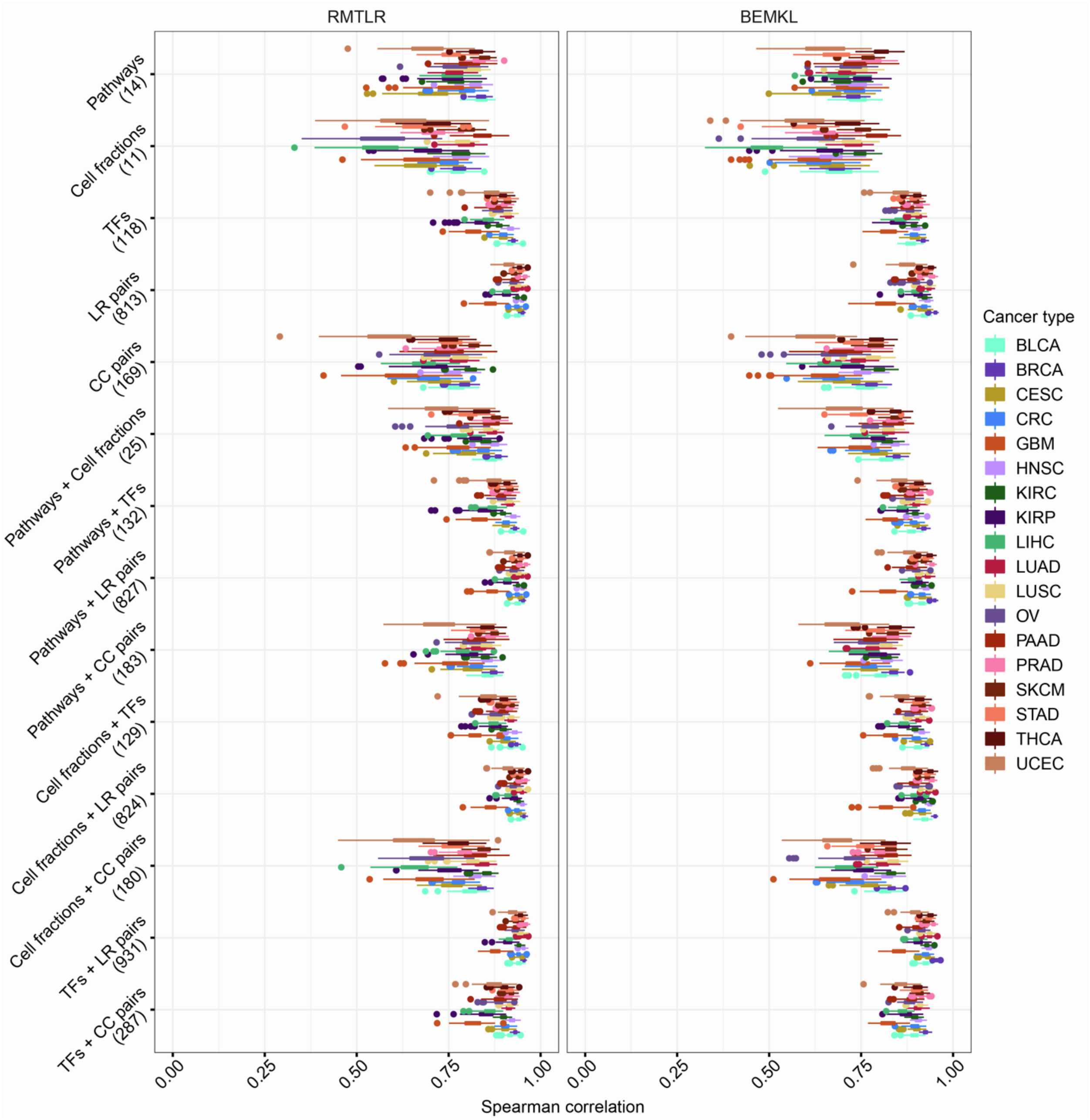

Figure S2. Randomized cross-validation performance of cancer-specific models trained on the TCGA patients. Models performances are shown for models trained separately for each system-based signature (single view) and for pairwise combinations of views. For each input data, the number of features is given in brackets.

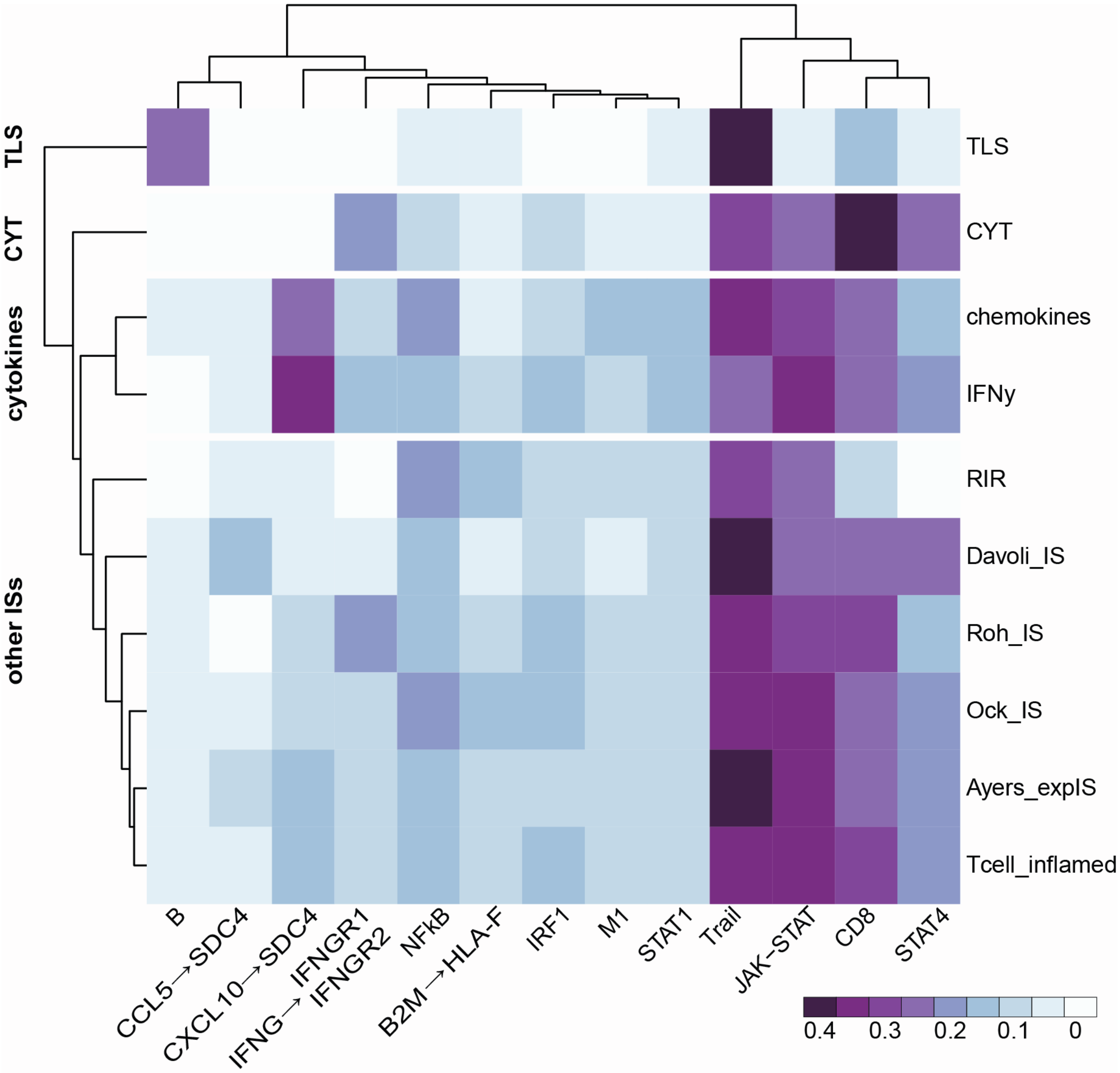

Figure S3. Heatmap showing the median across cancer types of the estimated weight of the features for each task. Only features with variance across tasks ≥ 0.0015 (top 1%) are shown in the heatmap. Tasks cluster in four main groups: 1. Tertiary lymphoid structures (TLS), 2. cytolytic activity (CYT), 3. cytokines related proxies (chemokines and IFNγ) , 4. all other immune signatures (ISs).

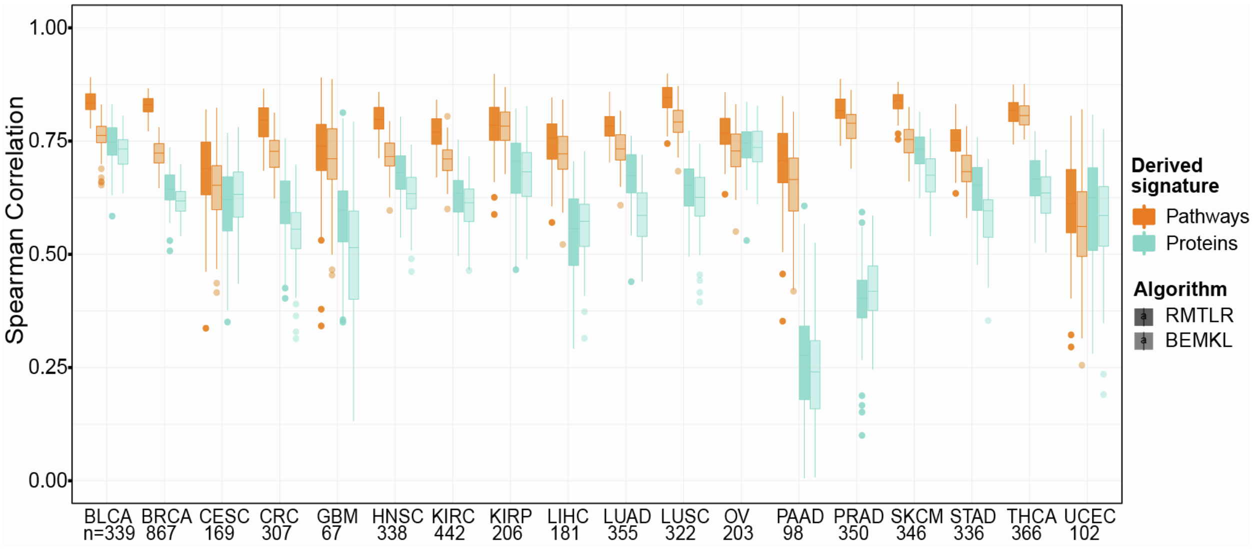

Figure S4. RMTLR and BEMKL cross-validation prediction performances for protein expression and pathway activity. Boxplots represent the distribution of prediction performances across the 100 randomized cross-validations on the TCGA data. The prediction performance is evaluated using Spearman correlation.

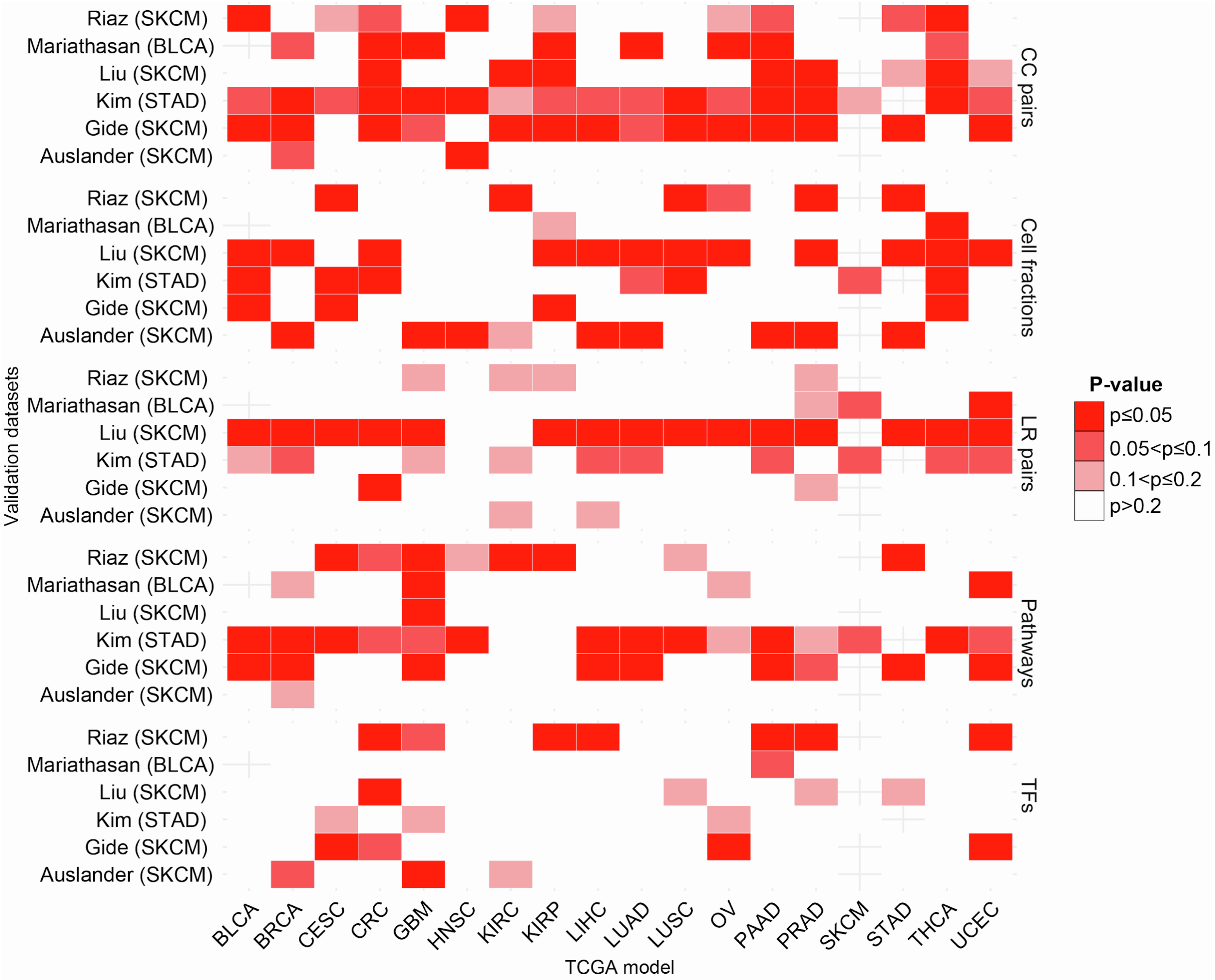

Figure S5. Comparison of prediction performance of cancer-type-specific models. The heatmap shows the p-value of the one-sided Wilcoxon-rank sum test used to assess if performances (AUC across tasks) obtained using the cancer-specific model for each dataset are better than performances obtained using the remaining 17 cancer-specific models (columns). This comparison was done for each view (different panels) and for each of the six datasets with known response to ICB (rows).

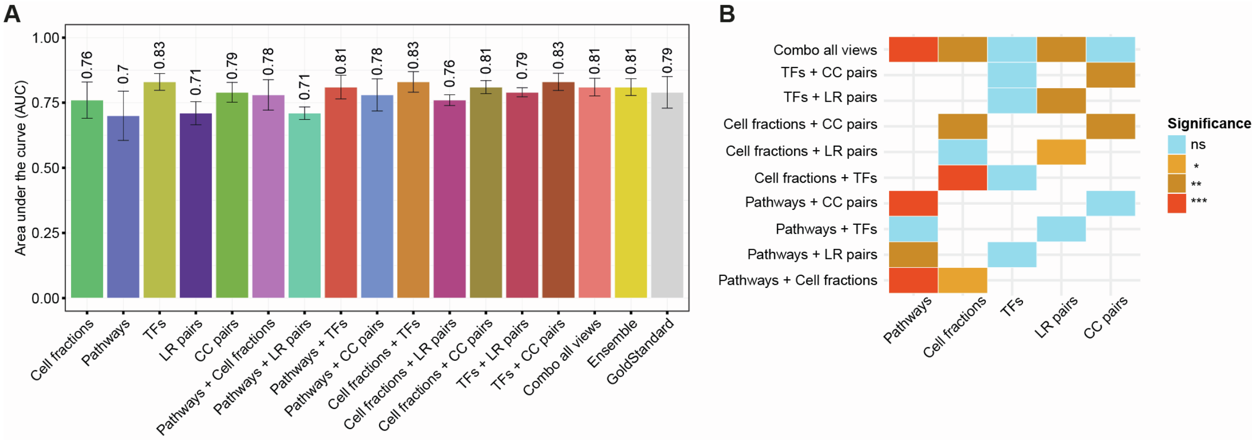

Figure S6. Prediction performances using BEMKL algorithm on the Auslander and Gide cohorts. (A) Area Under the Curve (AUC) values. Barplots represent the average AUC across tasks and error bars describe the corresponding standard deviation. (B) Performance comparison between single (x axis) and combined (y axis) views (one-side Wilcoxon signed-rank test). Statistical significance is indicated by colors according to the legend. The significant level (*p-value < 0.05, **p-value < 0.01, ***p-value < 0.001) indicates whether combining views improves the performance.

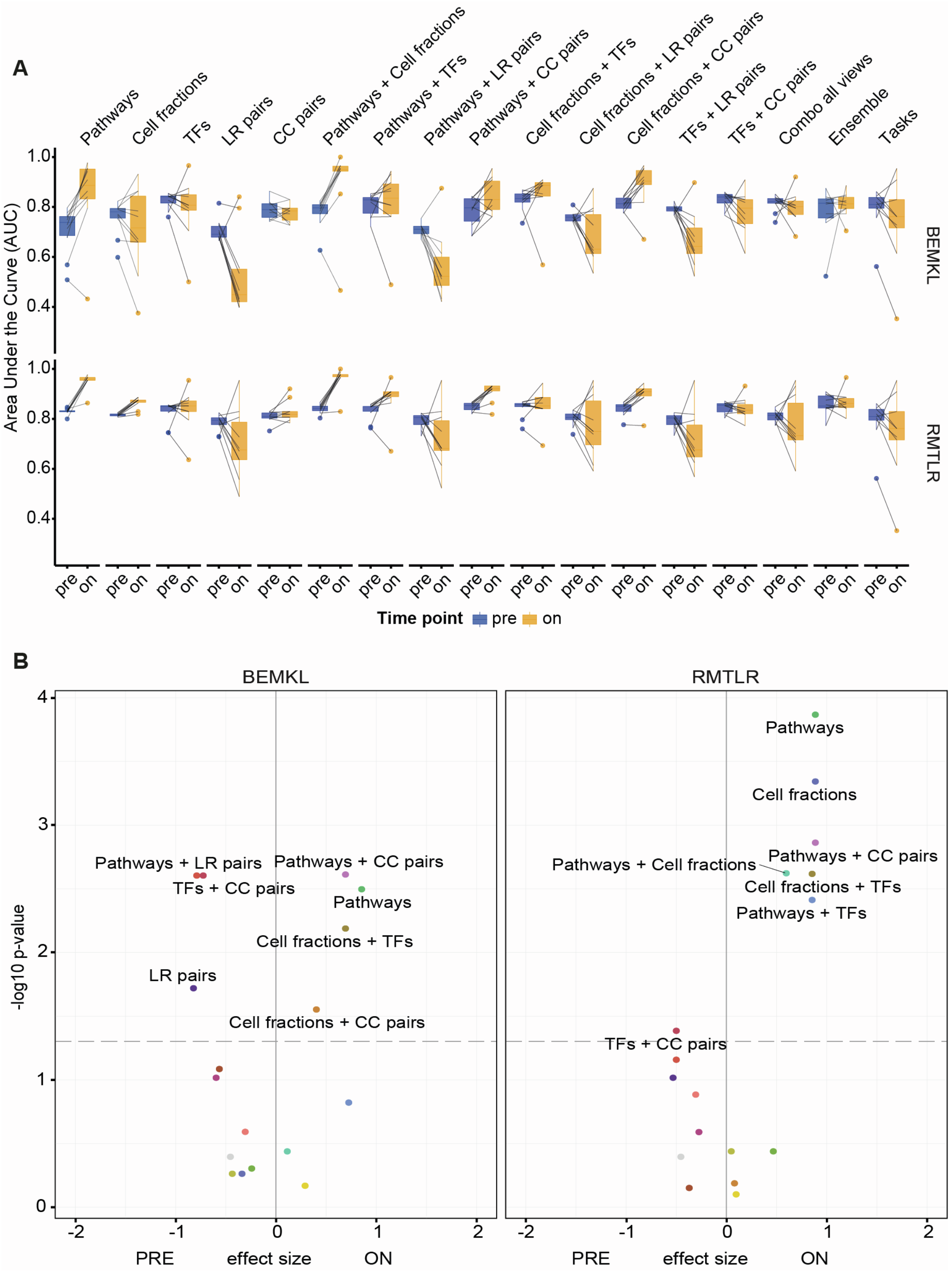

Figure S7. Comparison pre- vs on- treatment. (A) Comparison of Area Under the Curve (AUC) values for the different tasks computed on the pre-treatment (PRE) and on-treatment (ON) samples for the melanoma dataset. [1,2] Performances are shown for single views, pairwise combinations of views, combination of all views, average of single views predictions (ensemble), and all tasks (gold standard). (B) Volcano plots showing the statistical comparison of pre- vs on-treatment samples (two-sided Wilcoxon-rank sum test). Results are shown for both BEMKL and RMTLR algorithms.

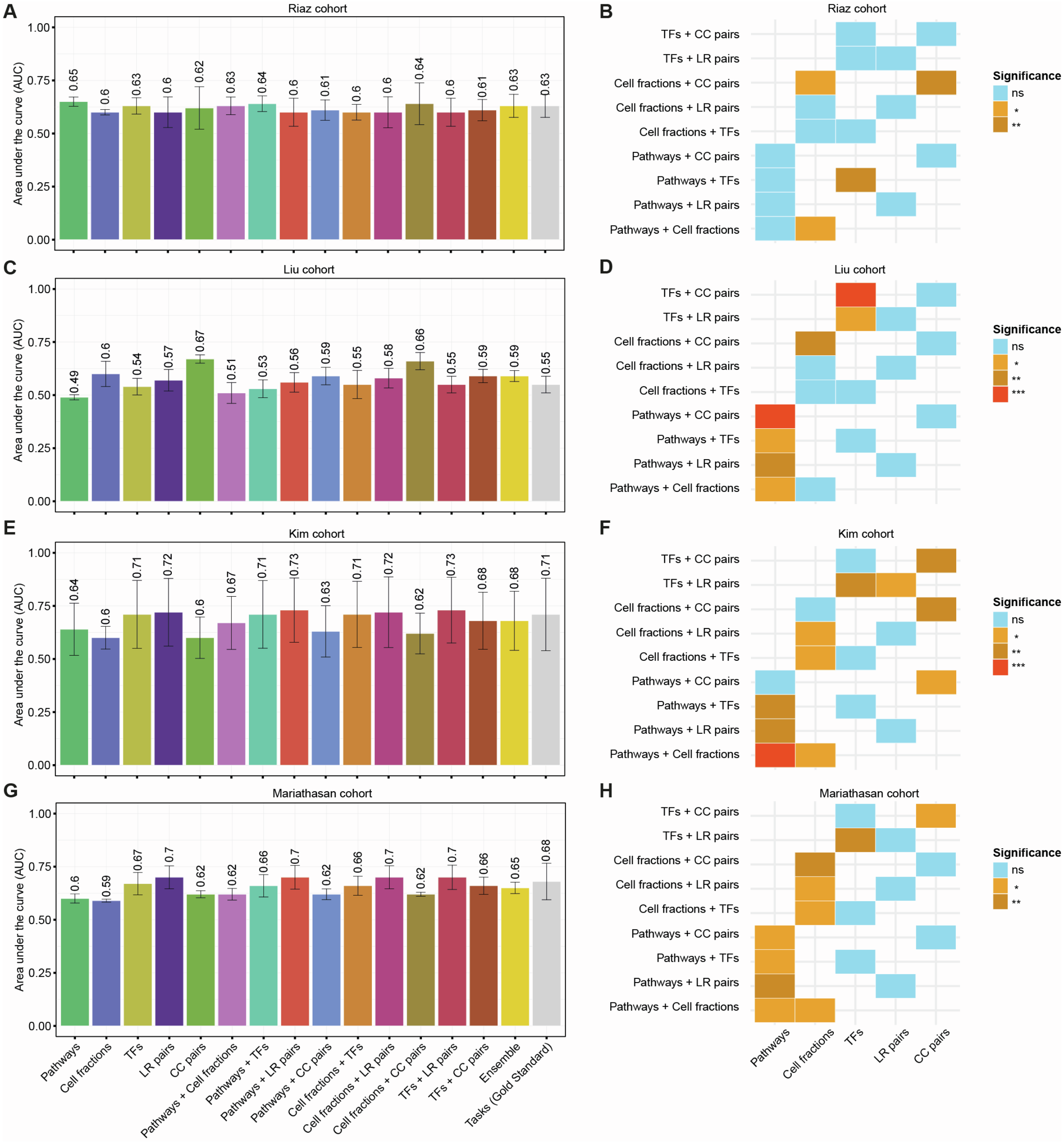

Figure S8. Overview of performances on the Riaz, Liu, Kim, and Mariathasan validation cohorts. Area Under the Curve (AUC) values for the (A) Riaz, (C) Liu, (E) Kim and (G) Mariathasan cohorts using the RMTLR algorithm. Performance comparison between single (x axis) and combined (y axis) views for (B) Riaz, (D) Liu, (F) Kim and (H) Mariathasan cohorts (one-side Wilcoxon signed-rank test). Statistical significance is indicated by colors according to the legend. The significant level (*p-value < 0.05, **p-value < 0.01, ***p-value < 0.001) indicates whether combining views improves the performance.

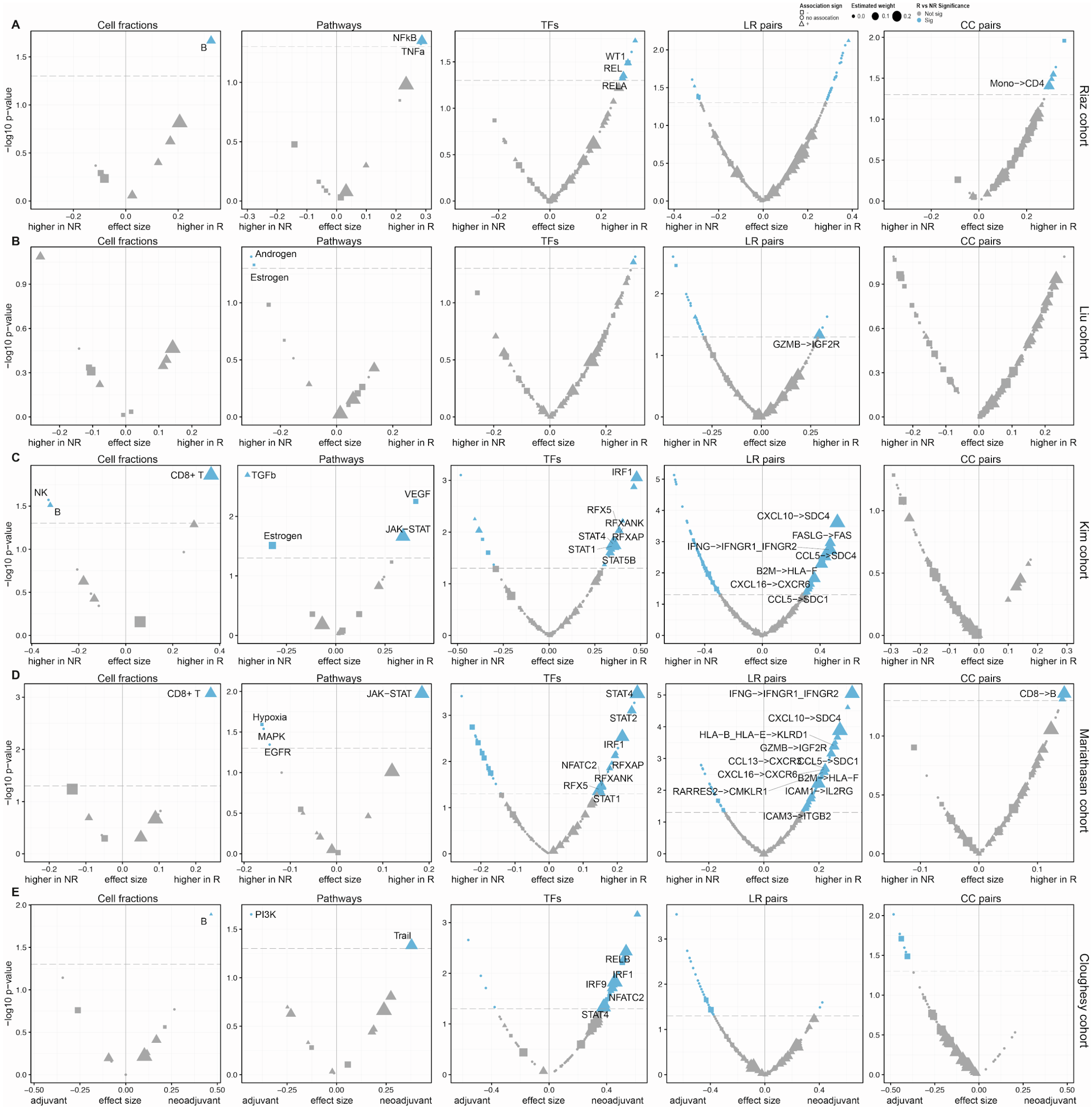

Figure S9. Comparison of biomarkers in responders vs non-responders. Volcano plots for systems biomarkers of the immune response from the (A) Riaz, (B) Liu, (C) Kim, (D) Mariathsan and (E) Cloughesy cohorts comparing non-responder (NR) and responder (R) patients. In the Cloughesy cohort, volcano plots compare systems biomarkers in adjuvant vs neoadjuvant anti-PD-1 therapy patients (two-sided Wilcoxon-rank sum test). Significant biomarkers (p-value < 0.05) are shown in blue. Biomarkers are drawn according to their corresponding sign (shape) and weight (size) obtained during model training. Labels are reported for the top 15 cancer-specific biomarkers *(based on the association with the tasks)* that are significantly different between R and NR.

SUPPLEMENTAL TABLES

Table S5. Overview of validation datasets.

Original study	Cancer type	TMB available	Prior therapies	Biopsy	Samples used	R and NR	RNA-seq fastq files
Auslander [1]	Melanoma (Metastasis)	NO	Therapy naive	Fresh-frozen	PD-1:<br>- Pre: n=9 (R=1, NR=8)<br>- On: n=17 (R=0, NR=17)	As reported in publication.	BioProject ID: PRJNA476140
Gide [2]	Melanoma (Metastasis)	NO	BRAFi	FFPE	PD-1:<br>- Pre: n=41 (CR=4, PR=15, PD=16, SD=6)<br>- On: n=9 (CR=0, PR=4, PD=4, SD=1)	R= CR, PR<br>NR= SD, PD	BioProject ID: PRJEB23709
Kim [3]	Gastric cancer (Metastasis)	YES	Prior failure of at least 1 line of chemotherapy (platinum)	Fresh-frozen	Pre: n=45 (CR=3, PR=9, PD=18, SD=15)	R= CR, PR<br>NR= SD, PD	BioProject ID: PRJEB25780
Riaz [4]	Advanced melanoma	YES	Nivo naive<br>Nivo prog	FFPE	Pre: n=45 (CR=3, PR=7, PD=23, SD=16)	R= CR, PR<br>NR= SD, PD	BioProject ID: PRJNA356761
Liu [5]	Melanoma (Metastasis)	YES	Prior MAPKTx<br>Therapy naive	FFPE	Pre: n=46 (CR=9, MR=1, PR=10, PD=19, SD=7)	R= CR, PR<br>NR= SD, PD	Supplementary files of the study
Mariathasan [6]	Bladder cancer	YES	Platinum based chemotherapy<br>Therapy naive	FFPE	Pre: n=192 (CR=25, PD=167)	R= CR<br>NR= PD	IMVigor210Biologies R package
Cloughesy [7]	Recurrent glioblastoma	NO	Previous first-line therapy with at least radiotherapy	Fresh-frozen	Pre: n=28<br>adjuvant anti-PD-1 = 15<br>neoadjuvant anti-PD-1 = 13	NA	BioProject ID: PRJNA498500

FFPE: Formalin-fixed paraffin-embedded
CR: Complete Responder; PR: Partial Responder; PD: Progressive Disease; SD: Stable Disease
R: Responder; NR: Non-responder

Table S6. List of cells included in the TME network.

Aggregate cell type	Cells in the literature	Cell types in network	Derivation
**Adipocytes**	Adipocytes [8,9]	Mature Adipocyte<br>Adipocyte Omental	
**B-cells**	B-cell [10]<br>CD20 B-cell [9,11]<br>CD19 B-cell [9]	CD19+ B cells	
**CD4+ T cells**	CD4+ T helper cells [8–10]<br>CD4+ T regulatory cells [8,10]	CD4+ T cells<br>CD4+CD25+CD45RA+ naive regulatory T-cells<br>CD4+CD25+CD45RA- memory regulatory T-cells<br>CD4+CD25-CD45RA+ naive conventional T-cells<br>CD4+CD25-CD45RA+ memory conventional T-cells	CD4+ NR T cells<br>CD4+ MR T cells<br>CD4+ NC T cells<br>CD4+ MC T cells
**CD8+ T cells**	CD8+ cytotoxic T cells [8–11]	CD8+ T cells	
**Dendritic cells**	Dendritic cell [8–11]	Dendritic Plasmacytoid<br>Dendritic Monocyte Immature derived	Dendritic Monocyte Id
**Endothelial cells**	Endothelial cells [10,11]<br>Vascular endothelial cells [8]<br>Lymphatic endothelial cells [8]	Endothelial Microvascular<br>Endothelial Lymphatic	
**Fibroblasts**	Fibroblasts [8–11]	Fibroblast Lymphatic<br>Fibroblast Skin Normal	
**Macrophages**	M1 macrophage [8–12]<br>M2 macrophage [8–10,12]	Macrophage Monocyte derived	Macrophage Monocyte
**Mast Cells**	Mast cells [9–11]	Mast cells<br>Mast cells stimulated	
**Monocytes Myeloid**	Myeloid derived suppressor cells [10,11]<br>Monocytes [9,11,12]	CD14+ Monocytes<br>CD14+CD16+ Monocytes<br>CD14+CD16- Monocytes<br>CD14-CD16+ Monocytes	
**NK Cells**	Natural killer cells [8–11]<br>Natural killer T-cells [8]	NK cells	
**Neutrophils**	Neutrophils [8,11]	Neutrophils	

SUPPLEMENTAL REFERENCES

1. Auslander, N., Zhang, G., Lee, J.S., Frederick, D.T., Miao, B., Moll, T., Tian, T., Wei, Z., Madan, S., Sullivan, R.J., et al. (2018). Robust prediction of response to immune checkpoint blockade therapy in metastatic melanoma. Nat. Med. *24*, 1545–1549.

2. Gide, T.N., Quek, C., Menzies, A.M., Tasker, A.T., Shang, P., Holst, J., Madore, J., Lim, S.Y., Velickovic, R., Wongchenko, M., et al. (2019). Distinct Immune Cell Populations Define Response to Anti-PD-1 Monotherapy and Anti-PD-1/Anti-CTLA-4 Combined Therapy. Cancer Cell *35*, 238–255.e6.

3. Kim, S.T., Cristescu, R., Bass, A.J., Kim, K.-M., Odegaard, J.I., Kim, K., Liu, X.Q., Sher, X., Jung, H., Lee, M., et al. (2018). Comprehensive molecular characterization of clinical responses to PD-1 inhibition in metastatic gastric cancer. Nat. Med. *24*, 1449–1458.

4. Riaz, N., Havel, J.J., Makarov, V., Desrichard, A., Urba, W.J., Sims, J.S., Hodi, F.S., Martín-Algarra, S., Mandal, R., Sharfman, W.H., et al. (2017). Tumor and Microenvironment Evolution during Immunotherapy with Nivolumab. Cell *171*, 934–949.e16.

5. Liu, D., Schilling, B., Liu, D., Sucker, A., Livingstone, E., Jerby-Arnon, L., Zimmer, L., Gutzmer, R., Satzger, I., Loquai, C., et al. (2019). Integrative molecular and clinical modeling of clinical outcomes to PD1 blockade in patients with metastatic melanoma. Nat. Med. *25*, 1916–1927.

6. Mariathasan, S., Turley, S.J., Nickles, D., Castiglioni, A., Yuen, K., Wang, Y., Kadel, E.E., III, Koeppen, H., Astarita, J.L., Cubas, R., et al. (2018). TGFβ attenuates tumour response to PD-L1 blockade by contributing to exclusion of T cells. Nature *554*, 544–548.

7. Cloughesy, T.F., Mochizuki, A.Y., Orpilla, J.R., Hugo, W., Lee, A.H., Davidson, T.B., Wang, A.C., Ellingson, B.M., Rytlewski, J.A., Sanders, C.M., et al. (2019). Neoadjuvant anti-PD-1 immunotherapy promotes a survival benefit with intratumoral and systemic immune responses in recurrent glioblastoma. Nat. Med. *25*, 477–486.

8. Balkwill, F.R., Capasso, M., and Hagemann, T. (2012). The tumor microenvironment at a glance. J. Cell Sci. *125*, 5591–5596.

9. Wang, M., Zhao, J., Zhang, L., Wei, F., Lian, Y., Wu, Y., Gong, Z., Zhang, S., Zhou, J., Cao, K., et al. (2017). Role of tumor microenvironment in tumorigenesis. J. Cancer *8*, 761–773.

10. Fridman, W.H., Pagès, F., Sautès-Fridman, C., and Galon, J. (2012). The immune contexture in human tumours: impact on clinical outcome. Nat. Rev. Cancer *12*, 298–306.

11. Fridman, W.H., Zitvogel, L., Sautès-Fridman, C., and Kroemer, G. (2017). The immune contexture in cancer prognosis and treatment. Nat. Rev. Clin. Oncol. *14*, 717–734.

12. Richards, D.M., Hettinger, J., and Feuerer, M. (2013). Monocytes and Macrophages in Cancer: Development and Functions. Cancer Microenvironment *6*, 179–191.
